# Spatial overlap and temporal synchrony between guilds of insect hosts and parasitoids

**DOI:** 10.1111/1365-2656.70228

**Published:** 2026-02-10

**Authors:** Laura J. A. van Dijk, Robert M. Goodsell, Anders F. Andersson, Brian L. Fisher, Elzbieta Iwaszkiewicz‐Eggebrecht, Piotr Lukasik, Andreia Miraldo, Pablo Peña‐Aguilera, Fredrik Ronquist, Tomas Roslin, Ayco J. M. Tack

**Affiliations:** ^1^ Department of Bioinformatics and Genetics Swedish Museum of Natural History Stockholm Sweden; ^2^ Science for Life Laboratory, Department of Gene Technology KTH Royal Institute of Technology Stockholm Sweden; ^3^ Entomology California Academy of Sciences San Francisco California USA; ^4^ Madagascar Biodiversity Center Parc Botanique et Zoologique de Tsimbazaza Antananarivo Madagascar; ^5^ Institute of Environmental Sciences, Faculty of Biology Jagiellonian University Kraków Poland; ^6^ Biodiversity and Sustainability Solutions–BaSS Aveiras de Baixo Portugal; ^7^ Department of Ecology Swedish University of Agricultural Sciences Uppsala Sweden; ^8^ Organismal and Evolutionary Biology Research Programme, Faculty of Biological and Environmental Sciences University of Helsinki Helsinki Finland; ^9^ Department of Ecology, Environment and Plant Sciences Stockholm University Stockholm Sweden

**Keywords:** climate change, feeding guilds, host–parasitoid dynamics, insect communities, land use, seasonality, spatiotemporal dynamics

## Abstract

1. How communities are structured into functional groups and trophic layers is key to understanding ecosystem functioning. Nonetheless, we lack insights about spatiotemporal variation in guild composition of communities and its causes.
2. To investigate spatial and temporal patterns and drivers of variation in insect feeding guilds, we combined data from a nationwide survey of Swedish insects using Malaise traps and DNA metabarcoding with a comprehensive trait database. We assigned species into one of three feeding guilds (phytophages, saprophages, predators) or into one of three associated parasitoid guilds. We then analysed patterns in species richness for each guild.
3. Species richness declined with latitude in all guilds. Beyond this gradient, local variation in species richness matched between hosts and their parasitoids. Yet, hosts and their parasitoids responded differently to habitat. The phenological peak of parasitoid species richness appeared later than the peak of their hosts, but the length of time lags varied among guilds. Spatiotemporal patterns were driven by guild‐specific responses to temperature, though much variation remained between seasons and locations even when controlling for temperature.
4. Overall, these patterns suggest that shifts in both climate and land use may alter the synchrony of insect trophic layers, with unknown consequences.

## Graphical abstract

This study identified a spatial overlap between insect host guild richness and parasitoid richness. Species richness in parasitoid guilds always increased later in the season than richness of their host guilds. These findings suggest that shifts in climate and land‐use may alter the synchrony of insect trophic layers.

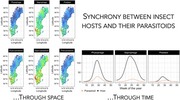

## 1 INTRODUCTION

Insects are the most taxonomically and functionally diverse animal clade (Stork, 2018; Wilson, 1987). They are also extremely sensitive to environmental pressures such as climate and land use changes (Millard et al., 2021; Outhwaite et al., 2022; Roslin, 2024; Wagner et al., 2021). To date, large‐scale descriptions of insects have been hampered by the diversity of their communities. Here, trait‐based functional groupings may offer a resolution. Instead of treating all insect species as unique, we impose structure on this diversity by delimiting groups of functionally similar species and analysing their responses (Wong et al., 2019). We will henceforth refer to such groups as functional guilds, that is, groups of organisms that exploit the same type of resource (Simberloff & Dayan, 1991). Insect guilds are intrinsically linked to key ecosystem functions (Tilman et al., 2014; Weisser & Siemann, 2013). Alterations to communities in terms of their functional composition (Damien & Tougeron, 2019; Forrest, 2016) may have dramatic consequences for ecosystem functioning (Eisenhauer et al., 2023; Prather et al., 2013). By resolving the responses of insect guilds to climate and land use, we may thus acquire a useful proxy for changes in functionally important aspects of animal communities.

Guilds can be defined from several different perspectives. The simplest division is between consumer and resource species, offering a partitioning into two trophic layers (Hanley & La Pierre, 2015). Another aspect is diet, with insects ranging from phytophages (plant feeders) to saprophages (detritus feeders) and predators (animal feeders) (Simberloff & Dayan, 1991). Each of these feeding guilds has its own parasitoids (Hawkins & Sheehan, 1994). Thus, the parasitoid of a predator occupies a third trophic layer, being indirectly linked to the primary consumers (herbivores or saprophages) through a predator. In this paper, we resolve insect communities into six feeding guilds based on diet and trophic position (phytophages, saprophages, predators and their corresponding parasitoids), which together span three trophic layers.

A large body of work linking insect traits to environmental responses shows that different guilds respond differently to environmental cues (Andrew & Hughes, 2004; Hermann et al., 2016; Newell et al., 2023; Voigt et al., 2007), which shape their spatial and temporal distributions (Andrew & Hughes, 2005; Forrest, 2016; Hodkinson et al., 1998; Lassau et al., 2005). Among relevant cues, temperature appears paramount—as ectotherms, insects are dependent on external heat sources (Price, 1997). Thus, flying insects emerge as temperatures rise at the beginning of the growing season (Gutiérrez & Wilson, 2021). Seasonal dynamics in response to temperature vary among insect taxa, depending, for example, on their life history traits (Forrest, 2016) and phenotypic plasticity (Hodgson et al., 2011). While temperature appears to be a main determinant of spatial and seasonal dynamics of insects, rainfall can also affect their spatiotemporal patterns, especially in the case of extreme precipitation events or droughts (Hermann et al., 2016; Newell et al., 2023). Besides climate, different habitat preferences among guilds can further dictate variation in their spatial distributions, causing differential responses to land use practices (van Dijk et al., 2024; Welti et al., 2022).

Beyond climatic and landscape effects, spatiotemporal dynamics of insect guilds can also be shaped by biotic interactions, such as the availability of food sources. While the spatiotemporal distribution of phytophages is strongly dictated by the timing of leaf‐out at the start of the season (Forkner et al., 2008), saprophages may rely on a more temporally constant resource (Rotheray, 2019). Predators are dependent on the emergence of the previous two guilds, whereas parasitoid guilds are dependent on their hosts for food. As parasitoids often specialise on a few host species, the distribution of parasitoids in space and time is expected to be tightly linked to host availability (Hassell, 2000). These effects should be most complex for parasitoids of predators due to their dependence on *two* lower trophic layers, with a direct dependence on predators and an indirect dependence on the prey of predators. These dependencies may cause them to emerge the latest in the season. Overall, we may expect guilds occupying the lowest trophic layer (phytophages and saprophages) to reach peak richness earliest in the season, followed by guilds occupying the intermediate trophic layer (predators and parasitoids of phytophages and saprophages) (Choi et al., 2010; Liu et al., 2000), and finally by guilds in the highest trophic layer (parasitoids of predators).

Current advances in molecular methods have made large‐scale invertebrate monitoring programs a reality (Buchner et al., 2025; Miraldo et al., 2025). However, a remaining impediment to assessing the dynamics of functional groups is the difficulty in designating functional guilds. This requires a healthy supply of taxonomic expertise and a knowledge of natural history, that is, what the organisms do and feed on. For many faunas, this task is extremely difficult, as most taxa are either unnamed, have unknown life histories, or both (Boyle et al., 2024; Ronquist et al., 2020; Stork et al., 2024). Nonetheless, for assemblages with a long history of study, these data are beginning to become available through compilation of existing data or targeted assessments by taxonomists (Ronquist et al., 2020).

Sweden has a nearly three centuries‐long history of taxonomy and natural history. In this study, we combined a dataset from a state‐of‐the‐art survey of Swedish insects (Miraldo et al., 2025) with a database of insect traits (Ronquist et al., 2020). Using these two datasets, we assessed how different functional guilds of insects differ in their spatial distribution and seasonal dynamics. We identified the taxa by DNA metabarcoding and used our trait data to assign all identified taxa into one of these six functional guilds: Phytophages, predators, saprophages and their corresponding parasitoids. Specifically, we answered the following questions:

1. What is the degree of spatial overlap between host guilds and their associated parasitoids?
2. What is the degree of temporal synchrony between host guilds and their associated parasitoids?
3. What are the environmental drivers of spatial overlap and temporal synchrony between host guilds and their associated parasitoids?

We hypothesised that hosts and their associated parasitoids will show a high degree of spatial overlap and temporal synchrony, where we expected a temporal delay in the emergence of parasitoids compared to hosts.

## 2 MATERIALS AND METHODS

In this study, we used Malaise traps to survey spatial and temporal patterns in the functional composition of insect guilds. While Malaise traps offer an efficient solution for sampling a large part of the insect community (Miraldo et al., 2025), they primarily sample flying insects. Thus, our measures of the timing of guild phenology are explicitly based on the timing of adult activity. As our first fundamental assumption, we thus presume that the timing of adult activity offers a useful proxy for the overall timing of the insects' life cycle. Second, we assume that Malaise samples provide constant detectability of insect groups over time. This does not necessitate that Malaise sampling would capture different taxa in exact proportion to their abundances (for counter‐evidence, see, e.g., Souto‐Vilarós et al., 2025), but simply that potential biases in detectability across taxa are constant in time and space. Third, our analyses are focused on species richness, not species‐ or guild‐specific abundances. This assumption is built on a simple consideration, that is, that the number of species detected at any one moment in time will reflect overall, guild‐specific activity, integrating across all taxa with an abundance above zero.

### 2.1 Sampling of insects

We use data from a spatially extensive insect survey comprising over 198 sampling locations across Sweden. Samples were collected using Malaise traps over the course of one full year (2019). Traps were collected weekly between April and October and monthly during the remainder of the year. Traps in locations with seasonally heavy snowfall were only sampled during months conducive to accessing and maintaining of the Malaise trap. The detailed sampling protocol and all aspects of the laboratory workflow and bioinformatics employed during DNA metabarcoding are given in Iwaszkiewicz‐Eggebrecht et al. (2023) and Sundh et al. (2024). Among the animal taxa resolved, we explicitly focused on arthropod taxa which primarily disperse by flight. Thus, we have excluded groups such as spiders and Collembola, which are richly represented in Malaise trap samples, but are likely to show deviant rates of capture due to their different modes of dispersal. Our study did not require ethical approval. For information on sampling permits, see Supporting Information Text S1.

### 2.2 Assignment of feeding guilds

Each OTU was designated as one of six categorical feeding guilds: *Phytophages, Predators, Saprophages, Phytophage parasitoid, Predator parasitoid* and *Saprophage parasitoid*. Feeding guilds were assigned based on trait data compiled from the scientific literature, as well as additional assessments by expert taxonomists. For the majority of species, data on the feeding guild were derived from Ronquist et al. (2020). These authors classified Swedish insects into one of six feeding guilds as based on their main feeding niche, defined as the niche of the immature stages (since they generally are the main feeding stages) (Ronquist et al., 2020). They first grouped the species into subfamilies for the three most species‐rich families (Ichneumonidae, Braconidae and Staphylinidae) and into families in all other cases. Expert taxonomists were then asked to assess the dominant feeding guild for each of these groups, that is, the guild likely applying to most of the species in the group. Following Ronquist et al. (2020), we then assume that all species in each taxonomic group are in the dominant guild. This allows us to generalise the guild data to the many insect species for which the life history is still unknown by capitalising on the fact that guild is usually a phylogenetically conserved trait (Peterson, 2011). For organisms missing a feeding guild designation from Ronquist et al. (2020), we derived a feeding guild based on additional literature sources (Hörren et al., 2022) or expert taxonomists. How these additional designations were made, and for which organisms, is detailed in the Supporting Information (Table S1).

### 2.3 Environmental data

To characterise potential drivers of spatiotemporal variation in the species richness of each guild, we focused on descriptors of climate, habitat and season (sensu week of the year). We used daily measurements (2019) of precipitation, as well as maximum and minimum temperature, downscaled to 1 km resolution from ERA5 climate estimates (Moreno & Hasenauer, 2016). We calculated the mean daily temperature as the mean of the maximum and minimum daily temperatures and then calculated the weekly mean temperature and precipitation from daily estimates. We compiled habitat data for 2019 from publicly available earth observation data sources. We used fractional cover estimates for 2019 from the Copernicus Land cover database (EU Copernicus Land Monitoring Service), downloaded at 100 m resolution. Cover estimates were extracted for tree cover (henceforth forest cover), shrubland, cropland and grassland. To provide an estimate of habitat type at a relevant scale for each trap location, we calculated the mean of each habitat cover value across the surrounding 1 km^2^ by taking the mean of the individual 100 m^2^ constituent pixels.

#### 2.3.1 Modelling the spatiotemporal dynamics of insect functional guilds

To analyse the impact of environmental drivers on the spatial distribution and seasonality of insect feeding guilds, we fitted hierarchical generalised additive models (HGAMS) to guild‐level species richness data (i.e. the total number of species within each guild). As feeding guilds may show non‐linear responses to environmental covariates, we fitted hierarchical generalised additive models (HGAMS) (Pedersen et al., 2019), implemented in the R package mgcv (Wood, 2001). These models accounted for non‐linear responses through the fitting of smooth functions of covariates and borrowed the ‘statistical strength’ of data rich groups to better estimate responses of groups with fewer observations. As data consisted of over‐dispersed counts of species (Poisson distribution), we modelled our response as a negative‐binomial distribution. We modelled the response of guild species richness to environmental covariates using a two‐level hierarchical model:

(1) $$\eta_{\mathrm{it}}=f_{s}\left(S_{t}\right)+f_{T}\left(T_{\mathrm{ti}}\right)+f_{P}\left(P_{\mathrm{ti}}\;\right)+\tau \left(X_{i},Y_{i}\right)$$

(2) $$y_{\mathrm{itg}}=\exp\left(\;\sum_{h=1}^{H}\beta_{\mathrm{hg}}x_{\mathrm{ih}}+f_{\mathrm{Sg}}\left(S_{t}\;\right)+f_{\mathrm{Tg}}\left(T_{\mathrm{ti}}\;\right)+f_{\mathrm{Pg}}\left(P_{\mathrm{ti}}\;\right)+\tau_{g}\left(X_{i},Y_{i}\right)+\mu_{\mathrm{ig}}+\gamma_{g}+\eta_{\mathrm{it}}\right)$$

Here, functional terms (f) represent smooth functions of different covariates included in the model. In Equation (1), these terms represent the overall (global) effect of these covariates on the linear predictor $\eta_{\mathrm{it}}$, where $f_{s}$ represents a smooth function for the global effect of week of the year at time *t* ($S_{t}$), $f_{T}$ the smooth effect of temperature $\left(T_{\mathrm{ti}}\right)$ at time *t* at site *i* and $f_{P}$ the smooth effect of precipitation ($P_{\mathrm{ti}}$) at time *t* and site *i*. The inclusion of the seasonal term $f_{s}\left(S_{t}\right)$ allowed us to account for unobserved time‐varying environmental variation not captured by temperature or precipitation covariates. The term $\tau \left(X_{i},Y_{i}\right)$ represents an interaction between two smooth effects over latitude (*Y*) and longitude (*X*), respectively; this term is included to account for unobserved spatial variation in the environment not captured by our other covariates. The term $\eta_{\mathrm{it}}$ therefore represents the global effects of climate, as well as broad‐scale seasonal and spatial variation in species richness $\left(y\right)$.

In Equation (2), guild‐level richness was modelled as a function of the global linear predictor and guild‐level covariates. We modelled the effect of the fractional cover of habitat covariate *h* as a function of *g*…*G* (*G* = 6) guild‐level random slopes. Therefore, $x_{\mathrm{ih}}$ is the cover value of habitat variable *h* at site *i*, and $\beta_{\mathrm{hg}}$ is the response of guild *g* to habitat covariate *h*. Group‐level seasonal and temperature effects are included through the smooth functions of covariates $f_{\mathrm{Sg}}\left(S_{t}\;\right)$, $f_{\mathrm{Tg}}\left(T_{i}\;\right)$ and $f_{\mathrm{Pg}}\left(P_{i}\;\right)$. The term $\tau_{g}\left(X_{i},Y_{i}\right)$ represents guild‐level spatial interaction terms between longitude (*X*) and latitude (*Y*), to account for large‐scale spatial variation in species richness between guilds that is not accounted for by geographic variation in our included habitat or climate covariates. Seasonal smooth effects $f_{\mathrm{Sg}}$ were modelled using cyclic cubic splines (*k* = 6) to ensure that seasonal trends start and end at the same time each year. All smooth climatic and spatial effects were modelled as thin plate regression splines (*k* = 6). The term $\mu_{\mathrm{ig}}$ is a trap‐level random effect for each guild to account for between‐site variability in species richness, and $\gamma_{g}$ is a random intercept for each guild, to account for baseline differences in species richness not accounted for in the covariates. As some guilds have relatively few observations, we assumed that each feeding guild behaves similarly to the global trend and parameterised the model so that the smoothing penalty term in group‐level smooths is shared with the global smooth (Pedersen et al., 2019). The result was that global and group‐level smooths share the same level of ‘wiggliness’, which determines the flexibility of the smooth terms. As traps were left for varying amounts of time across cold and warm seasons, we accounted for the effect of survey effort on species richness across guilds by including model offset terms that controlled for the exposure time of each trap across seasons (Shimadzu et al., 2016).

#### 2.3.2 Variable selection

As the model applied is computationally expensive, we used the double penalty approach to implement variable selection as described in Wood et al. (2011), using the ‘*select=TRUE*’, argument in the ‘*mgcv*::*gam*()’ function. This approach applies shrinkage to terms that contribute less to the models’ likelihood, effectively removing terms with small effect sizes and penalising model complexity (Marra & Wood, 2011). This approach allowed principled term selection while maintaining the predictive performance of the models, allowing us to fit a single model with all putative terms included.

#### 2.3.3 Spatial overlap between hosts and parasitoids

To compare the spatial trends in feeding guild richness, we simulated from the hierarchical model fitted in (1) and (2). We examined the guild‐level spatial distributions by estimating species richness across our environmental covariates. We made predictions from our model across a 1 km^2^ grid of environmental variables including the average temperature and precipitation in July, fractional habitat cover, and the spatial terms included in the model (Text S1, Figures S1–S6).

#### 2.3.4 Temporal synchrony between hosts and parasitoids

To investigate the seasonal dynamics across guilds, we simulated guild‐level trends in response to the combined effect of an annual temperature trend $\left(f_{T}\right)$, and the additional seasonal dynamics $\left(f_{s}\right)$. To achieve this, we estimated the average temperature trend across our sites by fitting a GAM to average weekly temperature values. We then simulated guild‐level trends by simulating across this trend for each individual guild. Subsequently, we examined guild‐specific phenology by comparing the week at which feeding guilds reached specific phenological phases (phenophases). We compared three phenophases: The start of the growing season for each guild, the peak of the season and the end of the season. We defined the start of the season as the first week in which the species richness of a guild reaches 10% of its maximum value plus the minimum guild‐specific richness (i.e. $SR^{\min}+\left({\mathrm{SR}}^{\max}/100\right)\times 10$). The season peak is defined as the maximum guild‐specific species richness $\left({\mathrm{SR}}^{\max}\right)$, and the season end is defined as the latest week in which the species richness of a guild reaches 10% of its maximum value plus the minimum guild‐specific richness. The minimum richness offset was included to account for guilds with different absolute richness values and variation in the shape of their seasonal trends. These phenophases do not specifically correspond to particular parts of community seasonal dynamics but provide a standardised measure to compare dynamics between guilds. We also compared the proportion of insects within each feeding guild at each of these phenophases to provide a measure of functional composition throughout the season.

#### 2.3.5 Drivers of spatial overlap and temporal synchrony between hosts and parasitoids

To identify drivers of spatial and seasonal variation in species richness of functional guilds, we directly examined model coefficients from the HGAM model described above. As climatic predictors, we examined the effects of short‐term fluctuations in weather conditions, including weekly average temperature and weekly average precipitation, on model predictions of species richness. As landscape predictors, we investigated the effect of fractional habitat cover, that is, the percentage cover of cropland, forest, grassland and shrubland. However, as all our habitat cover variables are proportional, they are inherently colinear—you cannot change the value of one without changing the value of another habitat cover variable. This means direct interpretation of model coefficients is difficult, as they might not be uniquely identifiable. Consequently, the fitted model may only accurately estimate variability in the response caused by the overall change in *habitat composition* from the observed data. We therefore selected scenarios that represent common habitat configurations (rather than changing each cover variable individually) in our observed data and simulated guild responses using our model. We selected four habitat scenarios (details on how we made this selection can be found in the appendix), which represented four habitat cover configurations commonly observed in our data (Table S2, Figure S2).

## 3 RESULTS

Overall, insect communities caught by Malaise traps were dominated by saprophagous species, followed by phytophages and then predators (Figure S7, Table S4). Saprophages were especially dominant during the start and end of the season. Parasitoids were generally less speciose than their hosts: Summed across host guilds and seasons, parasitoid species richness was 12% of host species richness (Figure S7a, Table S4). For saprophages and phytophages, the parasitoid: host ratio slowly built up over the season and then stagnated at the end of the season, whereas for predators, the parasitoid: host ratio was much more variable over time (Figure S8).

### 3.1 Spatial overlap between hosts and parasitoids

**FIGURE 1:**
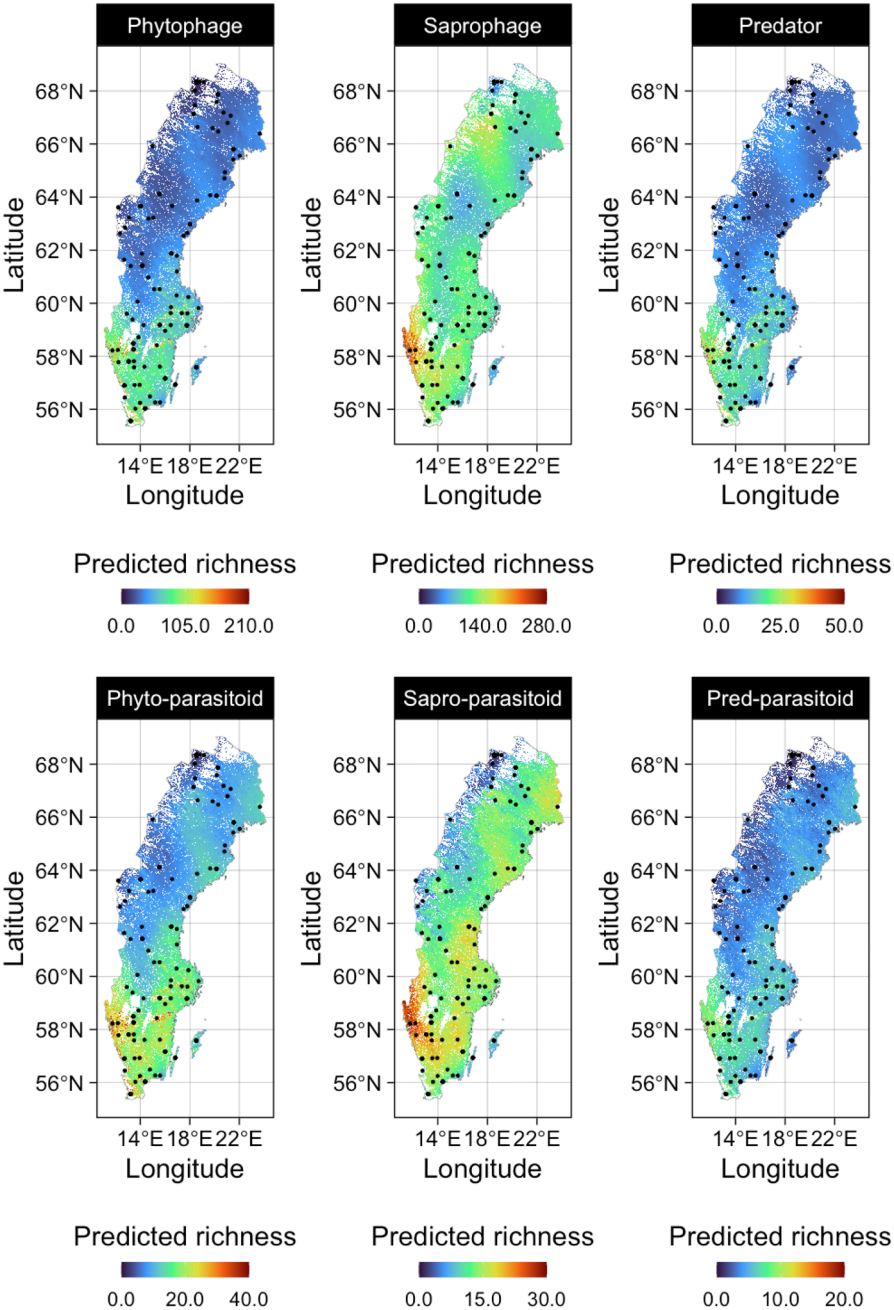
Spatial predictions of guild‐specific richness at a 10 km spatial resolution. Distributions were estimated across the average habitat cover for a 10 km grid and the mean July precipitation and temperature variables.

Species richness was generally predicted to be higher in the south compared to the north of Sweden for all functional guilds, with the lowest species richness in the northwest for all guilds except saprophagous insects (Figure 1). Beyond a general trend of decreasing species richness for higher latitudes, there was also pronounced local variation in species richness, not accounted for by the global pattern. Local variation in the spatial distribution of the richness of hosts (phytophages, predators and saprophages) was mirrored by their associated parasitoids (Figure 1). The only local spatial mismatch between host and parasitoid guilds was in the northwest (a mountainous area), where saprophages had a relatively high species richness compared to their associated parasitoids, which had the lowest species richness in this region.

### 3.2 Temporal synchrony between hosts and parasitoids

**FIGURE 2:**
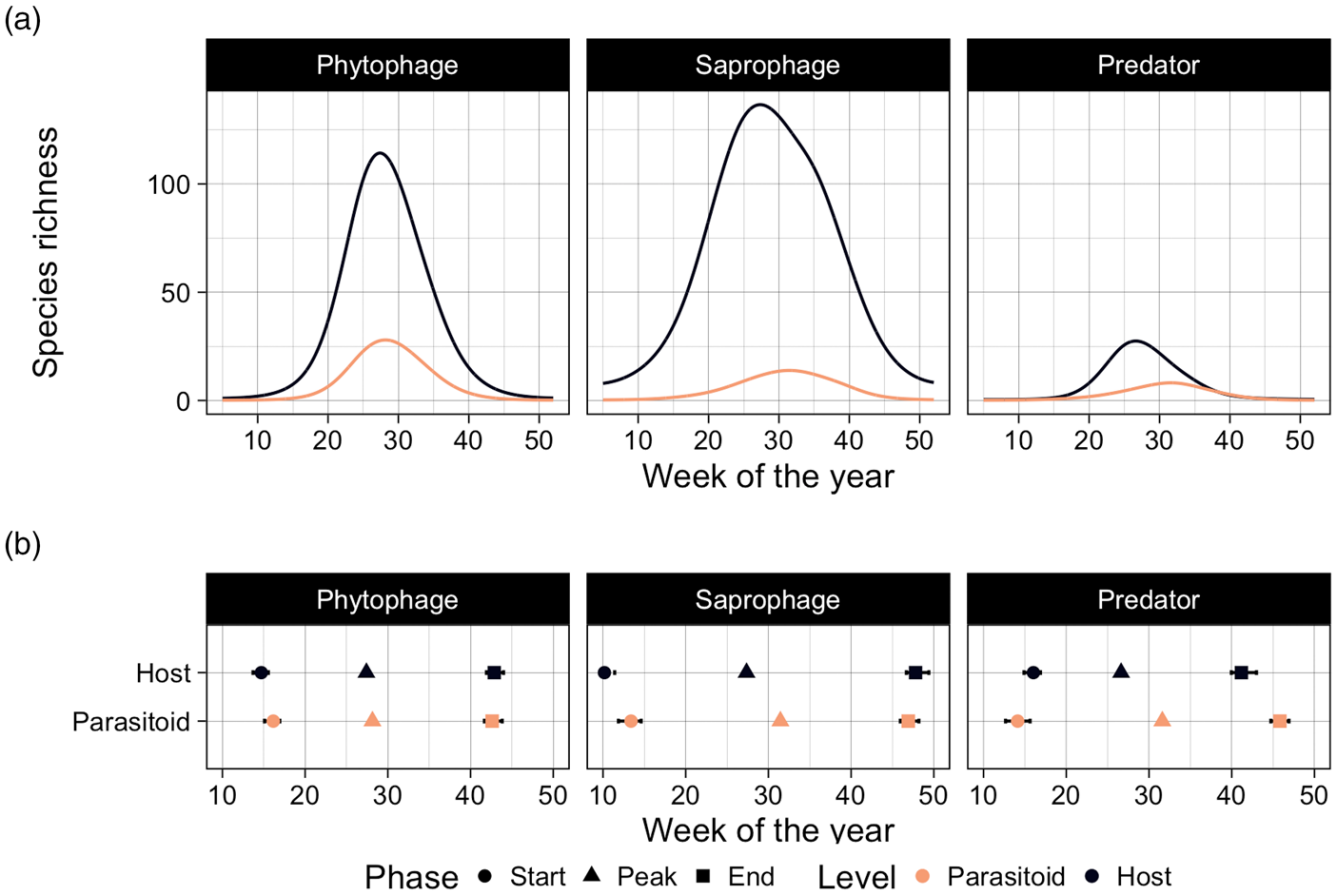
Temporal distribution of functional guilds. Panel (a) shows the temporal trends in each guild pair, estimated from the average yearly temperature trend. Coloured lines represent the host and parasitoid for each guild pair (phytophages, saprophages, predators and associated parasitoid communities). Panel (b) shows the week of different phenophases for each guild. The start of each season, the season peak (i.e. maximum guild‐specific species richness) and the end of the season for hosts and parasitoids in each guild pair. Error bars present 95% credible intervals calculated via MAP estimates of the mean.

While all functional guilds displayed a peak in species richness during the summer season, temporal synchrony between host and parasitoids in timing of the peak differed across guilds (Figure 2). Richness of saprophages and predators peaked first, followed by phytophages, then by parasitoids of phytophages, and finally by the richness of parasitoids of saprophages and predators (Figure 2). Species richness of host guilds *always* peaked earlier in the season than the richness of their corresponding parasitoids (Figure 2). The time lags between host and parasitoid richness peaks were much shorter for phytophages and their parasitoids (~1 week) compared to predators and saprophages and their parasitoid communities (~5.5 and ~4.5 weeks, respectively). For phytophages and saprophages, host richness decreased later in the season compared to parasitoid richness, while for predators, the timing of decrease in host richness overlapped with the decrease in parasitoid richness. Consequently, phytophages and saprophages had a much broader temporal distribution than their associated parasitoids, while the temporal distribution of predators and their parasitoids was more similar. Interestingly, the richness of saprophages also remained relatively high during the winter months compared to the richness of all other functional guilds, for which richness drops to zero in the off‐season (Figure 2).

### 3.3 Drivers of spatial overlap and temporal synchrony between hosts and parasitoids

**FIGURE 3:**
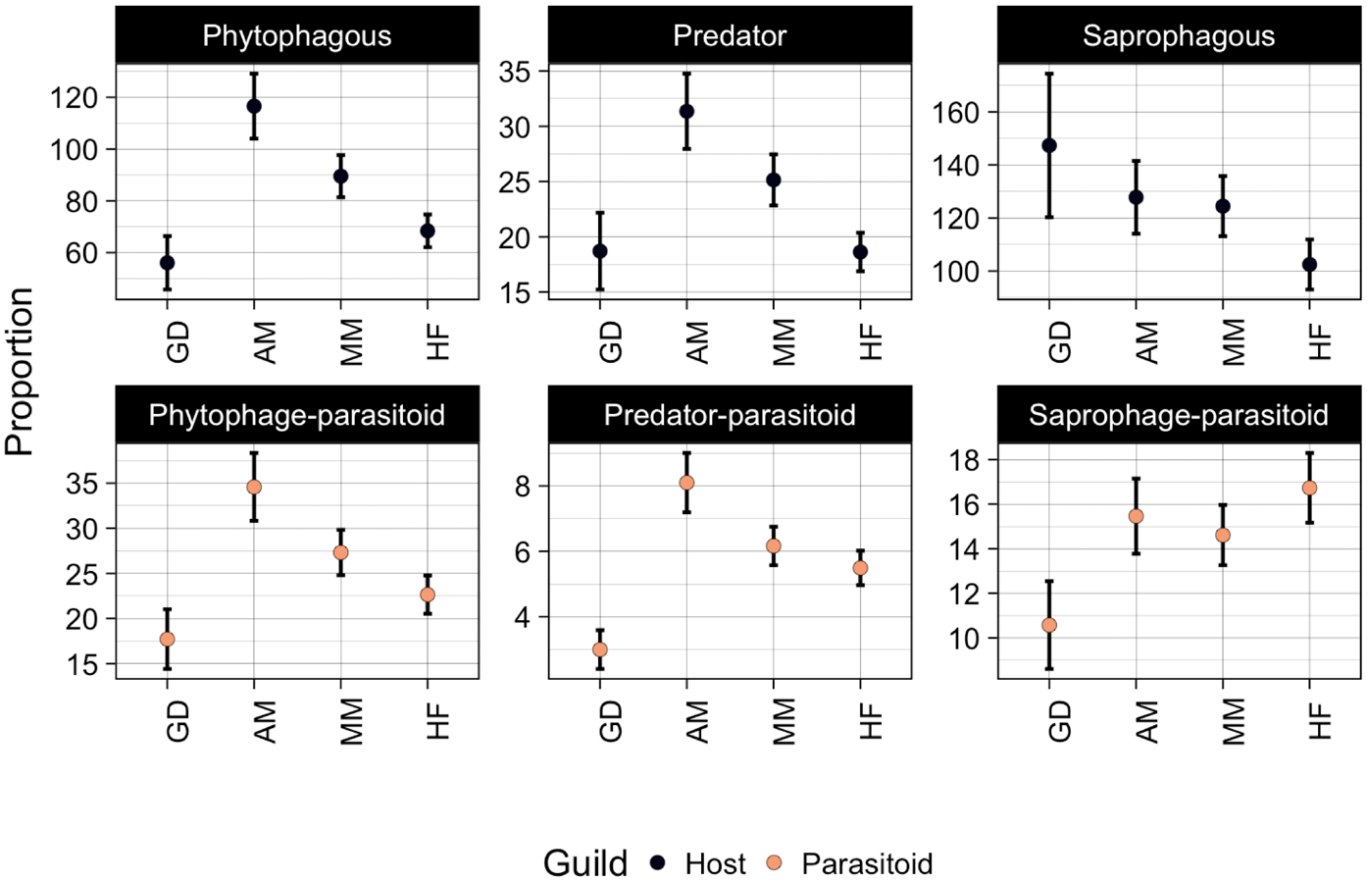
Guild‐specific species richness responses to different habitat configurations. GD represents a grass‐dominated landscape (crop = 0%, shrub = 24.7%, forest = 13.1%, grass = 60.7%), AM an agricultural mosaic (crop = 37.9%, shrub = 12.5%, forest = 23.8%, grass = 21.7%), MM a more even mixed mosaic (crop = 21.8%, shrub = 13.1%, forest = 35.2%, grass = 26.9%), and HF a heavily forested landscape (crop = 0.657%, shrub = 8.67%, forest = 76.2%, grass = 13.9%). Error bars present standard errors.

For all guilds except saprophages, habitat preference of host guilds was mirrored by the habitat preference of corresponding parasitoids (Figure 3). Phytophages, predators and their parasitoids reached their highest species richness in an agricultural mosaic landscape and the lowest in grass‐dominated landscapes. Saprophages and their parasitoids showed an inverse relationship in terms of habitat preferences (Figure 3): While saprophages tended to have the highest species richness in grass‐dominated landscapes and the lowest in heavily forested landscapes, their parasitoids showed the opposite pattern, with the lowest richness in grass‐dominated landscapes and a tendency for the highest richness in heavily forested landscapes.

**FIGURE 4:**
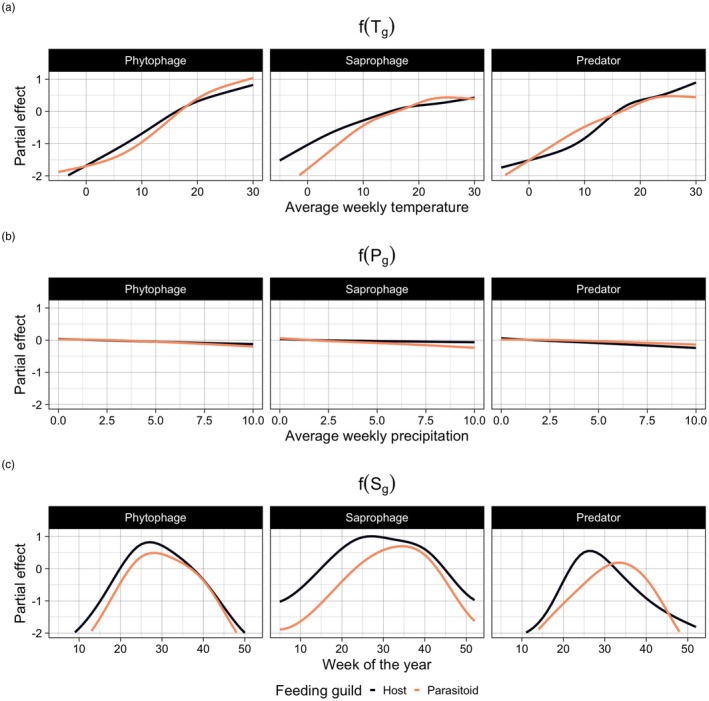
Climatic drivers of the distribution of functional guilds through space and time. The estimated smooth terms (global and guild‐specific) for the average weekly temperature (a), average weekly precipitation (b) and the seasonal component (c). The effect of a given predictor represents its effect after taking into account the effects of all other predictors included in the model (i.e. temperature, precipitation, week of the year and spatial location). Coloured solid lines represent the host and parasitoid for each guild pair (phytophages, saprophages, predators and associated parasitoid communities).

Overall, relatively warm weeks and sites showed higher guild‐specific species richness compared to relatively cold weeks and sites, though specific responses to temperature differed slightly among guilds (Figure 4a). Predators and the parasitoids of phytophages responded slower to relative increases in temperature compared to all other guilds. Also, after an initial positive effect, the effect of temperature on species richness levelled off for parasitoids of predators, as well as for saprophages and their associated parasitoids. Weekly precipitation had a negligible effect on the species richness of all functional guilds (Figure 4b), and as the expected degrees of freedom (Table S3) were low, this term was likely penalised out of the model via our variable selection approach. Lastly, there was a strong effect of the seasonal component on species richness of functional guilds through time (Figure 4c), indicating that seasonality of functional guilds could not be fully explained by the climatic and landscape predictors included in our model.

## 4 DISCUSSION

In this paper, we aimed to resolve spatiotemporal variation in the species richness of functional guilds among insects and their links to environmental drivers. Consistent with patterns across a wide set of taxa (Hillebrand, 2004; Kinlock et al., 2018), we found generally higher guild‐specific species richness at lower latitudes in all guilds. Nonetheless, guild‐specific richness showed pronounced variation around this trend, with a pronounced spatial overlap between host species richness and the richness of their parasitoids, though with a notable spatial mismatch between saprophages and their parasitoids. Hosts and their associated parasitoids generally showed similar habitat preferences, except saprophages and their parasitoids, which showed inverse preferences. In terms of temporal synchrony, species richness in parasitoid guilds always increased later in the season than species richness of their host guilds, though the length of this time lag differed among host‐parasitoid pairs. Temperature appeared as an important driver of spatial overlap and temporal synchrony for all functional guilds, with guild‐specific differences in responses.

### 4.1 The species richness of host and parasitoid guilds overlap in space

Overall, insect communities across Sweden were dominated by saprophagous species, followed by phytophages and predators. Parasitoid species richness was typically lower than host richness, and for predators, the species richness of parasitoids exceeded the richness of hosts in the end of the season. For an extended discussion on the functional composition of insect communities, see Text S2.

Across Sweden, which ranges ca 15° degrees in latitude, all host and parasitoid guilds showed a clear latitudinal gradient in species richness, with a high degree of spatial overlap between hosts and corresponding parasitoids. This latitudinal gradient was least pronounced for the richness of saprophages and their parasitoids. These guilds showed relatively higher species richness in the north compared to all other guilds. Hence, saprophagous species seem to be less sensitive to lower temperatures in the north compared to other guilds. This finding matches our finding on the relatively high species richness of saprophages during winter (see discussion below). Besides climatic effects, a less pronounced latitudinal gradient in richness of saprophages and their parasitoids could also be explained by their distinct ecology and diet compared to all other studied guilds. While phytophages and predators feed on *living* plants or animals, saprophages depend on dead organic matter and tend to have less specialised diets compared to other guilds (Maraun et al., 2003). Hence, as saprophages are less likely to be dependent on specific species that only occur at lower latitudes, the presence of saprophage species is likely to be less constrained by trophic bottom‐up effects compared to other guilds.

Besides large‐scale patterns, *local* variation in the spatial distribution of species richness (i.e. small scale *deviations* from the latitudinal pattern) showed strong spatial overlap between hosts and their associated parasitoids. Across guilds, the spatial distribution of phytophages and predators and their parasitoids showed more mutual resemblance compared to the spatial distribution of saprophages and their parasitoids. The spatial mismatch between saprophages and their parasitoids might be explained by differences in habitat preferences: The relatively low species richness of parasitoids of saprophages in the northwest, which consists of mountains with very low forest cover, may be attributable to their preference for heavily forested landscapes (see discussion below).

### 4.2 Time lags between host and parasitoid activity differ among guilds

Our results demonstrated that species richness peaked earlier in the season among hosts than their associated parasitoids and were thus slightly asynchronous in timing. This seems intuitive, since parasitoids depend on their host as a resource (Hawkins & Sheehan, 1994). Nonetheless, while host priority was repeated across all guilds, time lags varied among host–parasitoid pairs. In particular, the parasitoids of phytophages followed their host with a much shorter time lag than the parasitoids of predators and saprophages. Different time lags in different host–parasitoid guilds can be attributed to their lifestyles. As phytophages and their parasitoids are directly and indirectly dependent on plant resources, they will be faced with a brief time window for resources during leaf‐out in spring (Forkner et al., 2008). Predators and saprophages will typically be characterised by a wider diet and may thus be less constrained by timing to a given resource.

Regarding the end of the season, saprophages were the only guild where species richness did not decline to zero during winter. As saprophages feed on dead organic material, their food source will still be available during winter, and thus, some species may have adapted to withstand the harsher climatic conditions during winter (Block et al., 1990). Parasitoid richness on phytophages and saprophages decreased before the richness of their hosts, whereas predators were the only guild where species richness decreased simultaneously in hosts and parasitoids. Thus, a decrease in richness generally occurred earlier for parasitoid guilds than for their corresponding host guilds, consistent with patterns across increasing trophic positions. This was, however, not the case for predators (a second trophic level) and their parasitoids (a third trophic level), which decreased simultaneously at the end of the season. These results thus only partially confirm our hypothesised lag effect in the timing of emergence for parasitoid guilds relative to their host guilds, and more generally across increasing trophic positions. Overall, our results demonstrate that the seasonal dynamics differ among functional guilds and trophic levels.

### 4.3 Guild‐specific species richness varies with landscape and climate

As for the determinants of spatial overlap and temporal synchrony in species richness among insect guilds, both habitat and temperature emerged as important drivers. By comparison, precipitation had a limited effect on richness across all guilds.

Regarding responses to landscape composition, we found a remarkable similarity of species richness between hosts and their parasitoids among habitat configurations, except for saprophages and their parasitoids. Moreover, some habitat preferences were shared among guilds: All guilds except saprophages and their parasitoids reached their highest richness in agricultural mosaic landscapes, and the lowest in grass‐dominated and/or heavily forested landscapes. Even though van Dijk et al. (2024) showed that croplands had the highest insect biomass compared to other habitats in Sweden, we still expected to find a lower richness in agricultural landscapes due to severely decreased plant diversity (including monocultures) and the use of pesticides (Mancini et al., 2023) and fertilisers (Haddad et al., 2000). Nevertheless, Swedish agriculture uses relatively few pesticides (Gianessi et al., 2009) and is relatively extensive rather than intensive, with croplands immersed in heterogeneous landscapes (Figure S7). Moreover, open landscapes such as croplands may act as a flyway for insects between adjoining habitats (Cranmer et al., 2012). Surprisingly, saprophages and their parasitoids exhibited opposite preferences for habitat configurations, which matches with the low spatial overlap between these guilds, as discussed earlier. This suggests that landscape modification may decouple saprophages from their hosts, thus releasing them from top‐down control. Importantly, though, the current results explicitly concern species richness, not abundances, for which patterns may be different. In terms of richness, less parasitoids per host species translates to decreased vulnerability, with potential consequences for the stability of food webs and ecosystem functioning (Grass et al., 2018; Peralta et al., 2014).

The general effect of temperature is fully consistent with the dependence of insects as ectotherms on energy availability. Higher temperatures can speed development rates (Régnière et al., 2012), and as such, earlier emergence and flight times and higher abundances due to global warming are expected for many species (Buckley, 2022). Despite an overall positive response, responses to temperature varied across guilds. The estimated response of saprophages and their parasitoids to increasing temperature levelled off within the data range. Species richness of these guilds did not increase further when temperatures rose above a certain threshold, suggesting that further increases in temperature will yield no further gain in species richness for these guilds. Given that some saprophytic species were also active in winter at low temperatures, the highest temperatures may exceed the thermal tolerance of these species (Bale & Hayward, 2010). Richness of parasitoids of predators also levelled off in response to temperature, while the predator response did not, indicating a lower cutoff in the thermal tolerance of these parasitoids compared to their hosts (Shah et al., 2025). This is in line with previous studies, showing reduced development and survival with warmer temperatures (Moore et al., 2020), and a higher sensitivity to daily fluctuations in temperature (Moore et al., 2021), for parasitoids compared to their hosts. Lastly, predators as well as parasitoids of phytophages showed a shallower *initial* response to temperature compared to all other guilds.

Naturally, not all variation can be attributed to temperature, precipitation or habitat. The seasonal component of phenological change was captured by the effect of the week, which accounted for substantial variation. A species typical of the late summer will not fly in the early summer, no matter how warm it is, and vice versa. Likewise, sites will differ in many features not modelled here, with site‐to‐site variation leaving a substantial imprint.

### 4.4 What does flying species richness reflect?

Since Malaise traps primarily sample flying insects, our measurements of the timing of guild phenology are explicitly based on the timing of adult activity. Thus, our response variable reflects the number of species actively flying per guild during a given sampling period. To adopt this metric as a proxy for the overall timing of insect life cycles, we would assume that the timing of adult activity characterises the general phenology of the species. This assumption is generally true for predatory species, as they often predate both as adults and larvae, as well as for parasitoid species, which will typically attack the host as adults, selecting hosts at the egg or larval stage, then complete their larval stage on or within the host larva (Hawkins & Sheehan, 1994). This assumption is less clearly true for many phytophagous and saprophagous species, where it is often only the larvae which feed on plants or decaying matter (Price, 1997). However, even in these species, the peak of adult activity typically coincides with mating and egg laying, which represent a consistent and comparable checkpoint in life histories across guilds.

As our second main assumption, we presumed that the number of species detected at any one moment in time will reflect overall, guild‐specific activity. This assumption is easy to defend, since species richness will basically sum guild‐specific activity across all taxa with an abundance above zero. While some species will be represented by distinctly more individuals than others (Callaghan et al., 2023; Goodsell et al., 2025), species richness comes with another interpretation. In our study, consistent with our focus on guilds as functional units, species richness is represented by the number of species within a specific feeding guild active at any one time point. Since insects tend to be specialised on a relatively small set of resources (Forister et al., 2015; Hawkins & Sheehan, 1994), species richness presents an important metric of functional impact besides the abundance of individuals, which often strongly reflects a small set of species of limited resource range (Callaghan et al., 2023; Goodsell et al., 2025).

### 4.5 Caveats of this study

To reflect the functional roles of the larval stage of insect species, we split the insect fauna into a core set of six feeding guilds, as based on their main diet, including three host guilds and three parasitoid guilds. This choice was motivated by the fact that feeding traits are conservative enough that they can be reasonably assumed, in most cases, to be homogeneous within the family level groups we used (Peterson, 2011; Ronquist et al., 2020). However, this guild designation can naturally be further refined as based on innumerable other traits (Meier et al., 2024), on more specific modes of feeding (Shih et al., 2019), or on the life cycle characteristics of the adult life stages. Exactly what guilds are considered may naturally affect the conclusions and thus the scope for generalisations to other guild concepts. A second caveat is that, despite our reliance on expert taxonomists in the field, there remains some level of uncertainty in the assignment of species to guilds, especially so since taxa were assigned to guilds based on their taxonomic affinities at the (sub)family level. Overall, we note that trait data for insects (beyond Lepidoptera; Cerdeira‐Pérez et al., 2025; Shirey et al., 2022) are still poorly developed compared to trait data for, for example, plants (Báez et al., 2022; Kattge et al., 2011) and vertebrates (Herberstein et al., 2022; Huang et al., 2023; Oskyrko et al., 2024). Thus, we hope our study will contribute to and inspire a continued effort in the collection of trait data and the assignment of feeding guilds by expert taxonomists, ideally resulting in a comprehensive database covering all insect species and their life stages. A third caveat is the potential catching bias of Malaise traps for certain clades of insects, which could give a biased image of the actual representativeness of the different guilds in the natural community. While this may bias our results on observed community compositions, it should not interfere with our findings on the spatial and temporal patterns of feeding guilds. Moreover, to reduce this bias, we explicitly focused on flying insects alone (see Section 2). Still, catching bias remains a caveat hard to avert when attempting to sample a diverse community of insects, and no single sampling method will suffice to capture all insect taxa, see for example (Souto‐Vilarós et al., 2025). All in all, we conclude that our current results are conditional on the guild classification and sampling method used, but should not come with biases within this inference space.

### 4.6 Implications for global change

Overall, our results show that habitat and temperature drive the spatial overlap and temporal synchrony of species richness of functional guilds. Our findings come with key implications for predicting potential changes in the functionality of our ecosystems and imply that when conditions change, the functional composition of communities will change. Thus, shifts in both climate and land use may increase temporal asynchrony among trophic layers, with so far unknown consequences. In particular, shifts in interaction structure may occur, with direct impacts on host vulnerability and parasitoid generality. By highlighting these patterns and drivers of trophically related guilds through space and time, our findings contribute to predictions of potential shifts in the trophic structure of insect communities in response to climate and land use change—that is, identifying shifts which may disrupt the functionality of ecosystems.

## AUTHOR CONTRIBUTIONS

Ayco J.M. Tack, Tomas Roslin, Anders F. Anderson and Fredrik Ronquist conceived of the sampling design, and Andreia Miraldo and Elzbieta Iwaszkiewicz‐Eggebrecht coordinated the collection and processing of samples. The current approach and analyses were designed by Laura J.A. van Dijk, Robert M. Goodsell, Ayco J.M. Tack, Tomas Roslin and Fredrik Ronquist. Robert M. Goodsell and Laura J.A. van Dijk conducted the analyses and wrote the manuscript, with contributions from all authors.

## CONFLICT OF INTEREST STATEMENT

The authors have no conflicts of interest to declare.

## Supporting information


**Text S1.** Extended methods and results.
**Text S2.** Extended discussion on the composition of functional communities.
**Table S1.** Additional feeding guild designations of Insect families not classified in Ronquist et al., 2022.
**Table S2.** Habitat cover scenarios used to predict guild level responses to habitat variability. Each scenario represents a common configuration of different habitat types observed in the raw data.
**Table S3.** Coefficients from the fitted GAM. Here feeding niche is an alias for guild, and random effects are indicated via an Asterix*. Significant terms (*P*) are highlighted in bold.
**Table S4.** (a) Estimated Proportional and (b) absolute species richness at three time points during the year (week 10, 26 and 40, representing the start, peak and end of the growing season respectively). NB non‐integer values are seen due to the negative binomial response family in the model. See figure S7 for a visualization.
**Figure S1.** Observed versus predicted values from model fits for each guild. Dashed red line represents the 1:1 fit and the R‐squared values are provided for each individual guild.
**Figure S2.** An illustration of the changes of different habitat cover variables along PC1 of our habitat cover data decomposition (A), and the final cover configurations used in our habitat cover scenarios in our simulations of guild responses to habitat cover change (B).
**Figure S3.** Mean absolute error (MAE) and root mean square error (RMSE), for each feeding guild, calculated from 5‐fold cross validation. 5‐fold cross validation demonstrates relatively good predictive performance across guilds, with all guilds illustrating low variance in predictive error between folds. Guilds with higher Average richness's (Phytophages, and Saprophages), demonstrate higher predictive errors, but this is proportional considering the total richness of these guilds. Higher root mean error scores (RMSE) are present for most guilds, indicating that there are larger errors that are being penalised more severely.
**Figure S4.** The impact of spatially varying model terms on the species richness of Phytophagous insects (top row) and their parasitoids (bottom row). The figure displays predicted species richness for temperature, precipitation, the spatial interaction term, and habitat cover covariates. All non‐focal variables are set to 0, so that only the impact of the focal variable on the spatial patterns of guild species richness are visualised. Predictions for the temporally varying terms (precipitation and temperature), represent the average July values for those covariates. Habitat cover represents the sum of all habitat cover covariates included in the model.
**Figure S5.** The impact of spatially varying model terms on the species richness of Predatory insects (top row) and their parasitoids (bottom row). The figure displays predicted species richness for temperature, precipitation, the spatial interaction term, and habitat cover covariates. All non‐focal variables are set to 0, so that only the impact of the focal variable on the spatial patterns of guild species richness are visualised. Predictions for the temporally varying terms (precipitation and temperature), represent the average July values for those covariates. Habitat cover represents the sum of all habitat cover covariates included in the model.
**Figure S6.** The impact of spatially varying model terms on the species richness of Saprophagous insects (top row) and their parasitoids (bottom row). The figure displays predicted species richness for temperature, precipitation, the spatial interaction term, and habitat cover covariates. All non‐focal variables are set to 0, so that only the impact of the focal variable on the spatial patterns of guild species richness are visualised. Predictions for the temporally varying terms (precipitation and temperature), represent the average July values for those covariates. Habitat cover represents the sum of all habitat cover covariates included in the model.
**Figure S7.** The predicted composition of insect communities in terms of feeding guild richness throughout the year. The figure shows the proportion of organisms in each guild for all guild‐pairs(A), and across hosts only (B), and across parasitoids only (C) at three time points during the year representing the start (week 10), peak (week 26), and end (week 40) of the growing season. As all other predictors are kept constant, this figure represents the average effect across all sites. See Table S3 for numerical proportions for each of the guilds.
**Figure S8.** Parasitoid: host ratios of species richness for each of the guild‐pairs, at three time points during the year (week 10, 26 and 40, representing the start, peak and end of the growing season respectively). For raw proportional values of all guilds, see Table S3.
**Figure S9.** Three examples of traps placed next to croplands. In Sweden, agriculture is relatively extensive and croplands are intermixed with forests in the landscape.

## Data Availability

Data and code are available from Figshare https://doi.org/10.6084/m9.figshare.30086185 (van Dijk et al., 2026).
