# Supplementary material for: Spatial overlap and temporal synchrony between guilds of insect hosts and parasitoids
Source: J Anim Ecol. 2026 Feb 10;95(4):712–26. doi: 10.1111/1365-2656.70228 (PMC13039270; doi:10.1111/1365-2656.70228)
Supplement: Supplementary file 1 — Text S1. Extended methods and results. Text S2. Extended discussion on the composition of functional communities. Table S1. Additional feeding guild designations of Insect families not classified in Ronquist et al., 2022. Table S2. Habitat cover scenarios used to predict guild level responses to habitat variability. Each scenario represents a common configuration of different habitat types observed in the raw data. Table S3. Coefficients from the fitted GAM. Here feeding niche is an alias for guild, and random effects are indicated via an Asterix*. Significant terms (P) are highlighted in bold. Table S4. (a) Estimated Proportional and (b) absolute species richness at three time points during the year (week 10, 26 and 40, representing the start, peak and end of the growing season respectively). NB non‐integer values are seen due to the negative binomial response family in the model. See figure S7 for a visualization. Figure S1. Observed versus predicted values from model fits for each guild. Dashed red line represents the 1:1 fit and the R‐squared values are provided for each individual guild. Figure S2. An illustration of the changes of different habitat cover variables along PC1 of our habitat cover data decomposition (A), and the final cover configurations used in our habitat cover scenarios in our simulations of guild responses to habitat cover change (B). Figure S3. Mean absolute error (MAE) and root mean square error (RMSE), for each feeding guild, calculated from 5‐fold cross validation. 5‐fold cross validation demonstrates relatively good predictive performance across guilds, with all guilds illustrating low variance in predictive error between folds. Guilds with higher Average richness's (Phytophages, and Saprophages), demonstrate higher predictive errors, but this is proportional considering the total richness of these guilds. Higher root mean error scores (RMSE) are present for most guilds, indicating that there are larger errors that are being penalised m [file JANE-95-712-s001.docx]

**Supplementary material**

**Text S1.** Extended methods and results.

**Methods**:

*Feeding guild classifications:*

Where feeding guild classifications were missing for species in our sample, we attempted to designate a guild based on alternative sources. The families and number of constituent OTUs that we were able to designate are summarised in table **S1**. Any guild that did not have a guild designation was removed from the analysis.

*Selecting habitat cover scenarios*

Due to collinearity of proportional cover variables, sensible interpretation of model coefficients for our habitat terms is difficult. As habitat cover are proportional, and all values are therefore linearly dependent on each other, i.e. you cannot observe a change in one without a simultaneous change the others. To allow some ecologically meaningful interpretation of habitat cover responses in our analysis, we avoid the direct interpretation of cover coefficients and instead use model simulations of guild-specific responses to *combined* habitat cover variables. As there is multiple collinearity between habitat covariate data, care must be taken when conducting sensitivity analyses of this kind, as terms are likely not uniquely identifiable. Consequently, the fitted model may only accurately estimate variability in the response caused by the overall change in *habitat structure* from the observed data. We therefore select scenarios that represent common habitat configurations in our observed data and simulate guild responses using the original model. To select these scenarios, we decompose our raw proportional cover data using PCA and select the first axis (representing 54% of total variance) as an index of ‘overall habitat cover change’. We divided PC1 into even quantiles and used these as a guide from which to assess habitat cover change along our PC1 axis, we then select habitat cover proportions to represent four distinct scenarios indicative of real landscapes in our observed data.

Figure S2a, illustrates the change in our four habitat cover values along these quantiles, and S2b shows the final cover proportions of the final four habitat scenarios. These scenarios represent a i) grassland dominated landscape, with patches of forest and shrubland but no cropland ii) a cropland dominated agricultural mosaic, with some patches of forest, shrubland, and grassland, iii) a mixed mosaic with no clear dominant habitat type, iv) a heavily forested landscape with small patches of other habitats. These four habitat scenarios broadly align with commonly observed landscapes across Sweden.

*Model validation*

We ran two exercises to examine the predictive performance and stability of our model to assess its ability to provide valid inference. First, we simply plot observed vs predicted values for each guild (Figure S1). This exercise illustrated our models provided good fit to our data, without any evidence of systematic bias or error in our model predictions. Second, we perform an out of sample exercise, via k-fold (k=5) cross validation to further validate the model performance (Figure S3). Across all guilds we demonstrate very good levels of predictive performance. Parasitoid guilds illustrate higher levels of error compared to their species richness; however this is expected as they are less well sampled groups and will naturally display higher errors.

*Permits for sampling*

Samples at Stora Sjöfallets National park were collected under permit 5450-19 issued by the Swedish Environmental Protection Agency (Naturvårdsverket) and the Norrbotten county administrative board. All samples collected in Norrbotten county outside National parks were collected with permission from the National Property Board (Statens Fastighetsverk) and after consultation with all Sami villages concerned to decide the location of each Malaise trap. The remaining samples collected in this study were covered by Sweden´s right of access to private land (Allemansrätten) and did not necessitate a collection permit. More information about utilizing Swedish genetic resources can be found at the Swedish Environmental Protection Agency website: <https://www.naturvardsverket.se/en/guidance/species-protection/utilizing-genetic-resources>.

Samples in Madagascar were collected under research permits: 060/19/MEDD/SG/DGF/DSAP/SCB; 062/19/MEDD/SG/DGF/DSAP/SCB; 254/19/MEDD/SG/DGEF/DGRNE; 255/19/MEDD/SG/DGEF/DGRNE; 256/19/MEDD/SG/DGEF/DGRNE; 257/19/MEDD/SG/DGEF/DGRNE; 260/19/MEDD/SG/DGEF/DGRNE; 013/20/MEDD/SG/DGEF/DAPRNE; 060/20/MEDD/SG/DGEF/DAPRNE; 061/20/MEDD/SG/DGEF/DAPRNE; 062/20/MEDD/SG/DGEF/DAPRNE; 063/20/MEDD/SG/DGEF/DAPRNE; 064/20/MEDD/SG/DGEF/DAPRNE; 111/20/MEDD/SG/DGEF/DAPRNE; 112/20/MEDD/SG/DGEF/DAPRNE; 113/20/MEDD/SG/DGEF/DAPRNE; 114/20/MEDD/SG/DGEF/DAPRNE; 115/20/MEDD/SG/DGEF/DAPRNE.

All samples that were collected in Madagascar and then exported to Sweden for processing were exported under permits 096N-EA06/MG21 and 151N-EA09/MG2.

**Results**

*Sensitivity to spatial predictors*

Spatial patterns in guild richness are driven by a combination of all spatially varying terms in the model. However, across guilds some of the spatial terms contribute more to this overall pattern, for example the spatial term (which was included to account for unobserved environmental variability), contributes significantly more than other terms to the spatial variability in species richness across other guilds. This is as indicated by the large range of spatial variation in richness across guilds (Figure S2 – S4). Conversely precipitation illustrates relatively low levels of spatial variability, indicated by spatial homogeneity in the richness patterns. The impact of temperature and habitat cover varies strongly between guilds, for example, the average July temperatures illustrate large variation in spatial patterns for Saprophage and Predator parasitoids, whilst habitat cover more strongly influences the spatial pattern of Phytophages.

**Text S2**. Extended discussion on the composition of functional communities.

Overall, insect communities across Sweden were dominated by saprophagous species, followed by phytophages and predators. Parasitoid species richness was typically lower than host richness, and for predators, the species richness of parasitoids exceeded the richness of hosts in the end of the season.

In their overview of the Swedish insect fauna, Ronquist et al. (2020) estimated that half of the Swedish fauna consists of phytophages and their parasitoids, one-third of saprophages and their parasitoids, and one-sixth of predators and their parasitoids. Our estimates suggest a higher dominance of saprophages, with up to 83% of local communities consisting of saprophages and their parasitoids during the early season. We also demonstrated that the relative contributions of species richness among guilds remained similar over time, despite substantial variation in the species richness of the various guilds over the season. Thus, the relative richness of guilds at a national level is not directly reflected in their relative dominance at the level of local communities. What is more, parasitoid : host ratios among guilds differed between local communities (current study) and national richness patterns (Ronquist et al. (2020): Most strikingly, within local communities, the ratio of phytophage : parasitoid species was at most 1 : 0.23 (during peak season), while Ronquist et al. (2020) found a national ratio close to 1 : 1 between phytophages and their parasitoids. The discrepancy in guild-specific dominance between the national and the local level suggests dissimilar patterns of community dissimilarity, i.e. beta-diversity (Baselga, 2010), among taxa.

Through these estimates, the current study sheds new light on the functional composition of insect communities. This insight shows how previous challenges may be overcome through the combination of high-throughput sequencing with trait data. By efficiently characterising the species composition of communities, and assigning them traits from a joint database, we were able to describe the kinds of insects that comprise each group. This knowledge is essential, since contributions from functional guilds at various trophic levels will ultimately determine ecosystem functioning across space and time (Allan et al., 2015; Byrnes et al., 2014). Given the challenges in compiling functional trait data for abundant and hyper-diverse taxa (Voigt et al. 2007), and the challenges involved in large-scale sampling, studies to date have focused on specific taxonomic groups (Andrew and Hughes 2004, Cardoso et al. 2011) and on small spatial scales (Lassau et al. 2005, Gibb et al. 2006), but see (Guzman et al., 2021; Srivastava et al., 2023). Against this background, our contribution offers a new benchmark. While it is hard to monitor communities consisting of highly diverse taxa, of which most are rare (Goodsell et al. 2025), our study points to functional composition as a more accessible metric. We showed how we may derive a baseline description of functional composition – an exercise that may now be repeated to resolve changes over time (cf. Goodsell et al. 2025).

**Table S1.** Additional feeding guild designations of Insect families not classified in Ronquist et al., 2022.

| Class, Order, Family (number of OTUs) | Designation | Source |
| --- | --- | --- |
| \| Insecta \| Coleoptera \| *Apionidae* \| (21) \| \| --- \| --- \| --- \| --- \|  \| Insecta \| Coleoptera \| *Dryophthoridae* \| (1) \| \| --- \| --- \| --- \| --- \|  \| Insecta \| Coleoptera \| *Megalopodidae* \| (2) \| \| --- \| --- \| --- \| --- \|  \| Insecta \| Coleoptera \| *Rhynchitidae* \| (1) \| \| --- \| --- \| --- \| --- \|  \| Insecta \| Hemiptera \| *Adelgidae* \| (5) \| \| --- \| --- \| --- \| --- \|  \| Insecta \| Hemiptera \| *Aleyrodidae* \| (18) \| \| --- \| --- \| --- \| --- \|  \| Insecta \| Hemiptera \| *Aphalaridae* \| (12) \| \| --- \| --- \| --- \| --- \|  \| Insecta \| Hemiptera \| *Aphrophoridae* \| (15) \| \| --- \| --- \| --- \| --- \|  \| Insecta \| Hemiptera \| *Artheneidae* \| (1) \| \| --- \| --- \| --- \| --- \|  \| Insecta \| Hemiptera \| *Blissidae* \| (1) \| \| --- \| --- \| --- \| --- \|  \| Insecta \| Hemiptera \| *Cymidae* \| (2) \| \| --- \| --- \| --- \| --- \|  \| Insecta \| Hemiptera \| *Flatidae* \| (1) \| \| --- \| --- \| --- \| --- \|  \| Insecta \| Hemiptera \| *Fulgoridae* \| (1) \| \| --- \| --- \| --- \| --- \|  \| Insecta \| Hemiptera \| *Heterogastridae* \| (1) \| \| --- \| --- \| --- \| --- \|  \| Insecta \| Hemiptera \| *Liviidae* \| (4) \| \| --- \| --- \| --- \| --- \|  \| Insecta \| Hemiptera \| *Phylloxeridae* \| (2) \| \| --- \| --- \| --- \| --- \|  \| Insecta \| Hemiptera \| *Psyllidae* \| (30) \| \| --- \| --- \| --- \| --- \|  \| Insecta \| Hemiptera \| *Rhyparochromidae* \| (38) \| \| --- \| --- \| --- \| --- \|  \| Insecta \| Hemiptera \| *Thyreocoridae* \| (1) \| \| --- \| --- \| --- \| --- \|  \| Insecta \| Hemiptera \| *Triozidae* \| (19) \| \| --- \| --- \| --- \| --- \|  \| Insecta \| Hymenoptera \| *Masaridae* \| (6) \| \| --- \| --- \| --- \| --- \|  \| Insecta \| Lepidoptera \| *Bedelliidae* \| (1) \| \| --- \| --- \| --- \| --- \|  \| Insecta \| Lepidoptera \| *Crambidae* \| (56) \| \| --- \| --- \| --- \| --- \|  \| Insecta \| Lepidoptera \| *Epermeniidae* \| (4) \| \| --- \| --- \| --- \| --- \|  \| Insecta \| Lepidoptera \| *Erebidae* \| (49) \| \| --- \| --- \| --- \| --- \|  \| Insecta \| Lepidoptera \| *Lypusidae* \| (8) \| \| --- \| --- \| --- \| --- \|  \| Insecta \| Lepidoptera \| *Nolidae* \| (8) \| \| --- \| --- \| --- \| --- \|  \| Insecta \| Lepidoptera \| *Praydidae* \| (1) \| \| --- \| --- \| --- \| --- \|  \| Insecta \| Lepidoptera \| *Schreckensteiniidae* \| (1) \| \| --- \| --- \| --- \| --- \|  \| Insecta \| Lepidoptera \| *Scythrididae* \| (6) \| \| --- \| --- \| --- \| --- \|  \| Insecta \| Lepidoptera \| *Stathmopodidae* \| (1) \| \| --- \| --- \| --- \| --- \| | Phytophagous | SLU taxonomist – Tomas Roslin |
| \| Insecta \| Coleoptera \| *Dasytidae* \| (2) \| \| --- \| --- \| --- \| --- \|  \| Insecta \| Coleoptera \| *Malachiidae* \| (4) \| \| --- \| --- \| --- \| --- \|  \| Insecta \| Coleoptera \| *Melyridae* \| (18) \| \| --- \| --- \| --- \| --- \|  \| Insecta \| Coleoptera \| *Rhadalidae* \| (18) \| \| --- \| --- \| --- \| --- \|  \| Insecta \| Diptera \| *Atelestidae* \| (2) \| \| --- \| --- \| --- \| --- \|  \| Insecta \| Diptera \| *Chamaemyiidae* \| (31) \| \| --- \| --- \| --- \| --- \|  \| Insecta \| Diptera \| *Megamerinidae* \| (1) \| \| --- \| --- \| --- \| --- \|  \| Insecta \| Diptera \| *Ragadidae* \| (2) \| \| --- \| --- \| --- \| --- \|  \| Insecta \| Hymenoptera \| *Eumenidae* \| (11) \| \| --- \| --- \| --- \| --- \| | Predators | SLU taxonomist – Tomas Roslin |
| \| Insecta \| Blattodea \| *Blaberidae* \| (1) \| \| --- \| --- \| --- \| --- \| \| Insecta \| Blattodea \| *Blattidae* \| (5) \| \| Insecta \| Blattodea \| *Ectobiidae* \| (5) \| \| Insecta \| Coleoptera \| *Brentidae* \| (13) \| \| Insecta \| Coleoptera \| *Stenotrachelidae* \| (1) \| \| Insecta \| Dermaptera \| *Spongiphoridae* \| (1) \| \| Insecta \| Diptera \| *Bolitophilidae* \| (27) \| \| Insecta \| Diptera \| *Camillidae* \| (1) \| \| Insecta \| Diptera \| *Canthyloscelididae* \| (1) \| \| Insecta \| Diptera \| *Cylindrotomidae* \| (4) \| \| Insecta \| Diptera \| *Cypselosomatidae* \| (1) \| \| Insecta \| Diptera \| *Diastatidae* \| (12) \| \| Insecta \| Diptera \| *Ditomyiidae* \| (4) \| \| Insecta \| Diptera \| *Drosdophilidae* \| (5) \| \| Insecta \| Diptera \| *Heleomyzidae* \| (97) \| \| Insecta \| Diptera \| *Limoniidae* \| (208) \| \| Insecta \| Diptera \| *Opetiidae* \| (1) \| \| Insecta \| Diptera \| *Pediciidae* \| (16) \| \| Insecta \| Diptera \| *Tanypezidae* \| (1) \| \| Insecta \| Psocodea \| *Amphipsocidae* \| (1) \| \| Insecta \| Psocodea \| *Caeciliusidae* \| (27) \| \| Insecta \| Psocodea \| *Ectopsocidae* \| (5) \| \| Insecta \| Psocodea \| *Elipsocidae* \| (6) \| \| Insecta \| Psocodea \| *Epipsocidae* \| (7) \| \| Insecta \| Psocodea \| *Lachesillidae* \| (4) \| \| Insecta \| Psocodea \| *Liposcelididae* \| (7) \| \| Insecta \| Psocodea \| *Mesopsocidae* \| (6) \| \| Insecta \| Psocodea \| *Paracaeciliidae* \| (1) \| \| Insecta \| Psocodea \| *Peripsocidae* \| (7) \| \| Insecta \| Psocodea \| *Philotarsidae* \| (2) \| \| Insecta \| Psocodea \| *Psocidae* \| (17) \| \| Insecta \| Psocodea \| *Psyllipsocidae* \| (1) \| \| Insecta \| Psocodea \| *Stenopsocidae* \| (5) \| \| Insecta \| Psocodea \| *Trichopsocidae* \| (1) \| \| Insecta \| Psocodea \| *Trogiidae* \| (6) \|   Top of Form | Saprophagous | SLU taxonomist – Tomas Roslin |
| \| Insecta \| Coleoptera \| *Ripiphoridae* \| (1) \| \| --- \| --- \| --- \| --- \|  \| Insecta \| Diptera \| *Polleniidae* \| (12) \| \| --- \| --- \| --- \| --- \|  \| Insecta \| Hymenoptera \| *Azotidae* \| (1) \| \| --- \| --- \| --- \| --- \|  \| Insecta \| Hymenoptera \| *Dryinidae* \| (101) \| \| --- \| --- \| --- \| --- \|  \| Insecta \| Hymenoptera \| *Megastigmidae* \| (3) \| \| --- \| --- \| --- \| --- \|  \| Insecta \| Hymenoptera \| *Ormyridae* \| (3) \| \| --- \| --- \| --- \| --- \|  \| Insecta \| Hymenoptera \| *Orussusidae* \| (1) \| \| --- \| --- \| --- \| --- \|  \| Insecta \| Hymenoptera \| *Signiphoridae* \| (1) \| \| --- \| --- \| --- \| --- \| \| Insecta \| Hymenoptera \| *Mymarommatidae* \| (3) \| | Phytophage-parasitoid | SLU taxonomist – Tomas Roslin |
| \| Insecta \| Diptera \| *Phaeomyiidae* \| (1) \| \| --- \| --- \| --- \| --- \| | Saprophage-parasitoid | SLU taxonomist – Tomas Roslin |
| Insecta Coleoptera Melyridae (18)  Insecta Diptera Atelestidae (2) | Predator | Hörren et al 2022. |
| Insecta Coleoptera Anobiidae (16) | Phytophagous | Hörren et al 2022. |

**Table S2.** Habitat cover scenarios used to predict guild level responses to habitat variability. Each scenario represents a common configuration of different habitat types observed in the raw data.

| **Scenario** | **Description** | **Crop (%)** | **Shrub (%)** | **Forest (%)** | **Grass (%)** |
| --- | --- | --- | --- | --- | --- |
| **GD** | Grass-dominated landscape | 0.0 | 24.7 | 13.1 | 60.7 |
| **AM** | Agricultural mosaic | 37.9 | 12.5 | 23.8 | 21.7 |
| **MM** | Mixed mosaic | 21.8 | 13.1 | 35.2 | 26.9 |
| **HF** | Heavily forested landscape | 0.657 | 8.67 | 76.2 | 13.9 |

**Table S3.** Coefficients from the fitted GAM. Here feeding niche is an alias for guild, and random effects are indicated via an Asterix*. Significant terms (*P*) are highlighted in bold.

| **Term** | **edf** | **Ref.df** | **F** | ***P*** |
| --- | --- | --- | --- | --- |
| s(feeding_niche, forest_cover)* | 3.0678 | 6 | 326.335 | **0.131150** |
| s(feeding_niche, crop_cover)* | 5.4917 | 6 | 3389.798 | **1.58e-06** |
| s(feeding_niche, shrub_cover)* | 2.9279 | 6 | 101.483 | 0.125150 |
| s(feeding_niche, grass_cover)* | 4.5275 | 6 | 576.429 | **0.002345** |
| s(feeding_niche)* | 2.1448 | 5 | 0.853 | **< 2e-16** |
| s(week_year) | 3.7337 | 4 | 14.057 | **< 2e-16** |
| s(week_year, feeding_niche) | 22.0555 | 29 | 167.061 | **< 2e-16** |
| s(mean_w_temp) | 2.9431 | 5 | 13.647 | **< 2e-16** |
| s(mean_w_temp, feeding_niche) | 23.7974 | 35 | 30.157 | **< 2e-16** |
| s(mean_w_prec) | 0.9563 | 5 | 4.339 | **< 2e-16** |
| s(mean_w_prec, feeding_niche) | 9.3984 | 35 | 1.061 | **2.57e-05** |
| s(longitude_wgs84, latitude_wgs84) | 24.7929 | 29 | 334.068 | **< 2e-16** |
| s(longitude_wgs84, latitude_wgs84, feeding_niche) | 20.3276 | 179 | 45.308 | **0.000215** |
| s(trap_id, feeding_niche)* | 947.6614 | 1093 | 13.976 | **< 2e-16** |

**Table S4. (a)** Estimated Proportional and **(b)** absolute species richness at three time points during the year (week 10, 26 and 40, representing the start, peak and end of the growing season respectively). NB non-integer values are seen due to the negative binomial response family in the model. See figure S7 for a visualization.

**(a)**

| **Week** | **Guild pair** | **Host** | **Parasitoid** |
| --- | --- | --- | --- |
| 10 | Saprophage | 0.80 | 0.03 |
| 10 | Phytophage | 0.11 | 0.01 |
| 10 | Predator | 0.03 | 0.01 |
|  |  |  |  |
| 26 | Saprophage | 0.431 | 0.0323 |
| 26 | Phytophage | 0.352 | 0.0815 |
| 26 | Predator | 0.0869 | 0.0165 |
|  |  |  |  |
| 40 | Saprophage | 0.676 | 0.0694 |
| 40 | Phytophage | 0.162 | 0.0374 |
| 40 | Predator | 0.0261 | 0.0293 |

**(b)**

| **Week** | **Guild** | **Host** | **Parasitoid** |
| --- | --- | --- | --- |
| 10 | Saprophage | 13.99 | 0.48 |
| 10 | Phytophage | 1.98 | 0.25 |
| 10 | Predator | 0.45 | 0.22 |
|  |  |  |  |
| 26 | Saprophage | 134.8 | 10.09 |
| 26 | Phytophage | 110.2 | 25.50 |
| 26 | Predator | 27.2 | 5.16 |
|  |  |  |  |
| 40 | Saprophage | 63.47 | 6.51 |
| 40 | Phytophage | 15.17 | 3.51 |
| 40 | Predator | 2.45 | 2.75 |


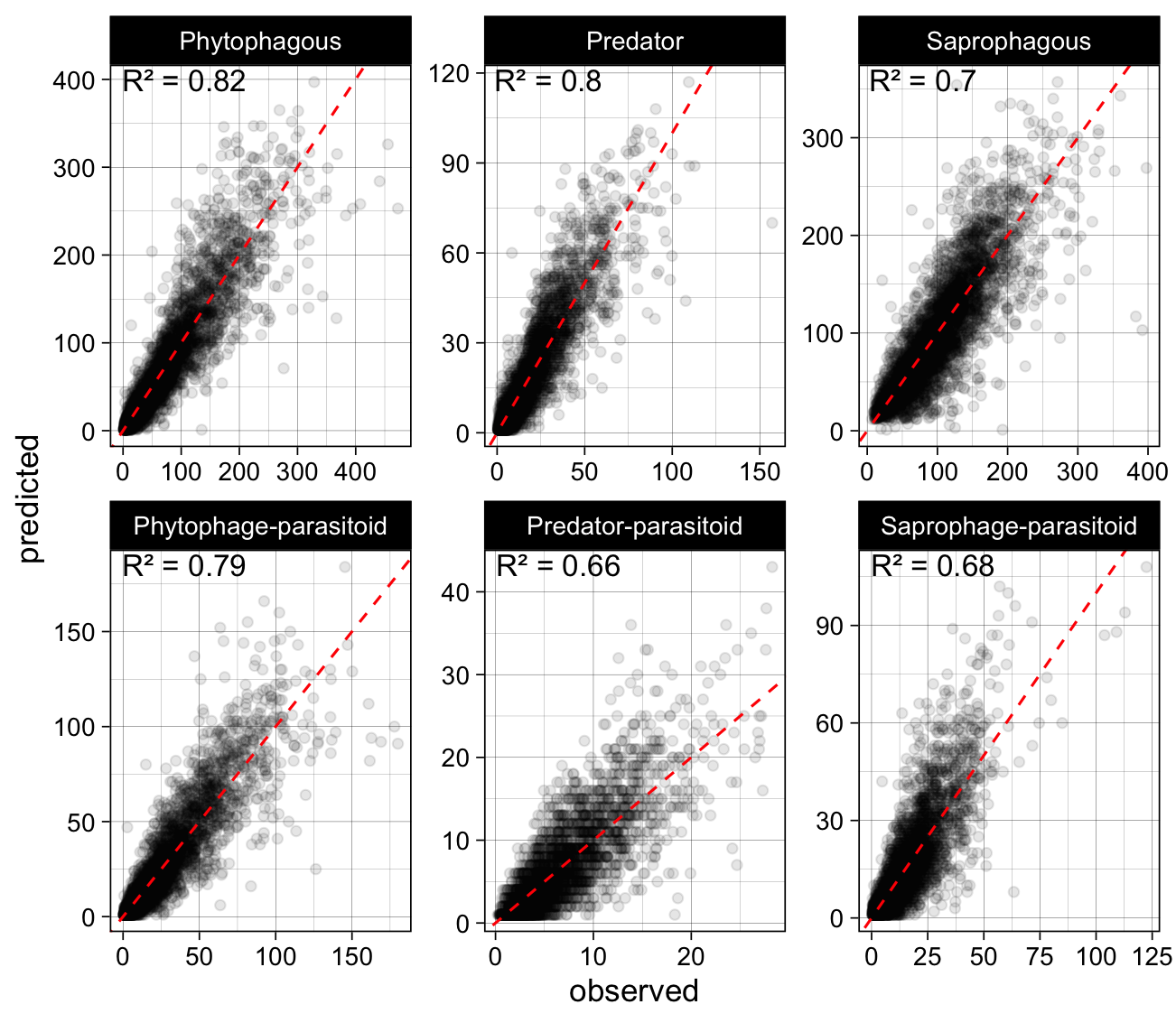


**Figure S1**. Observed vs predicted values from model fits for each guild. Dashed red line represents the 1:1 fit and the R-squared values are provided for each individual guild.

**
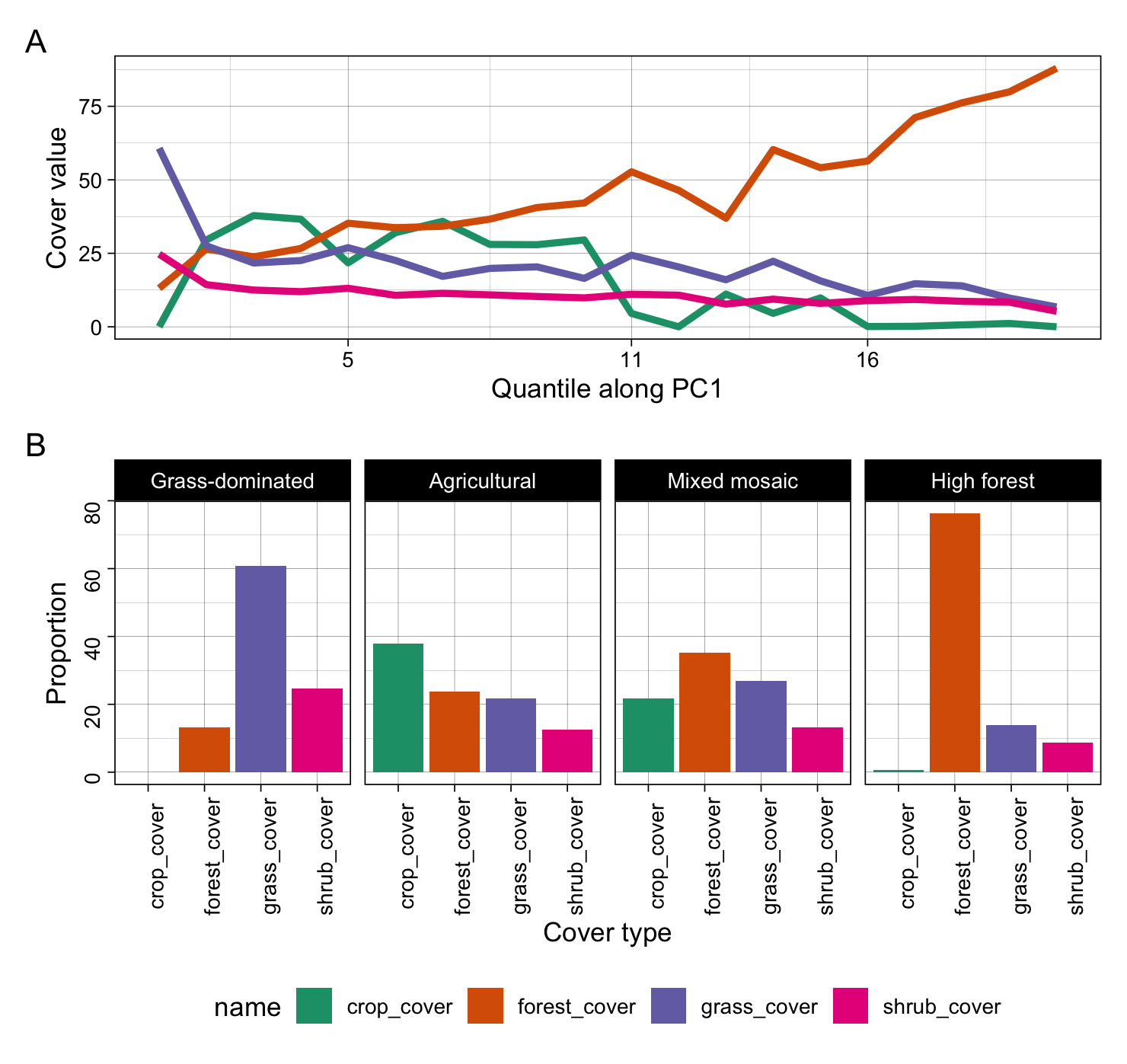
**

**Figure S2.** An illustration of the changes of different habitat cover variables along PC1 of our habitat cover data decomposition (A), and the final cover configurations used in our habitat cover scenarios in our simulations of guild responses to habitat cover change (B).


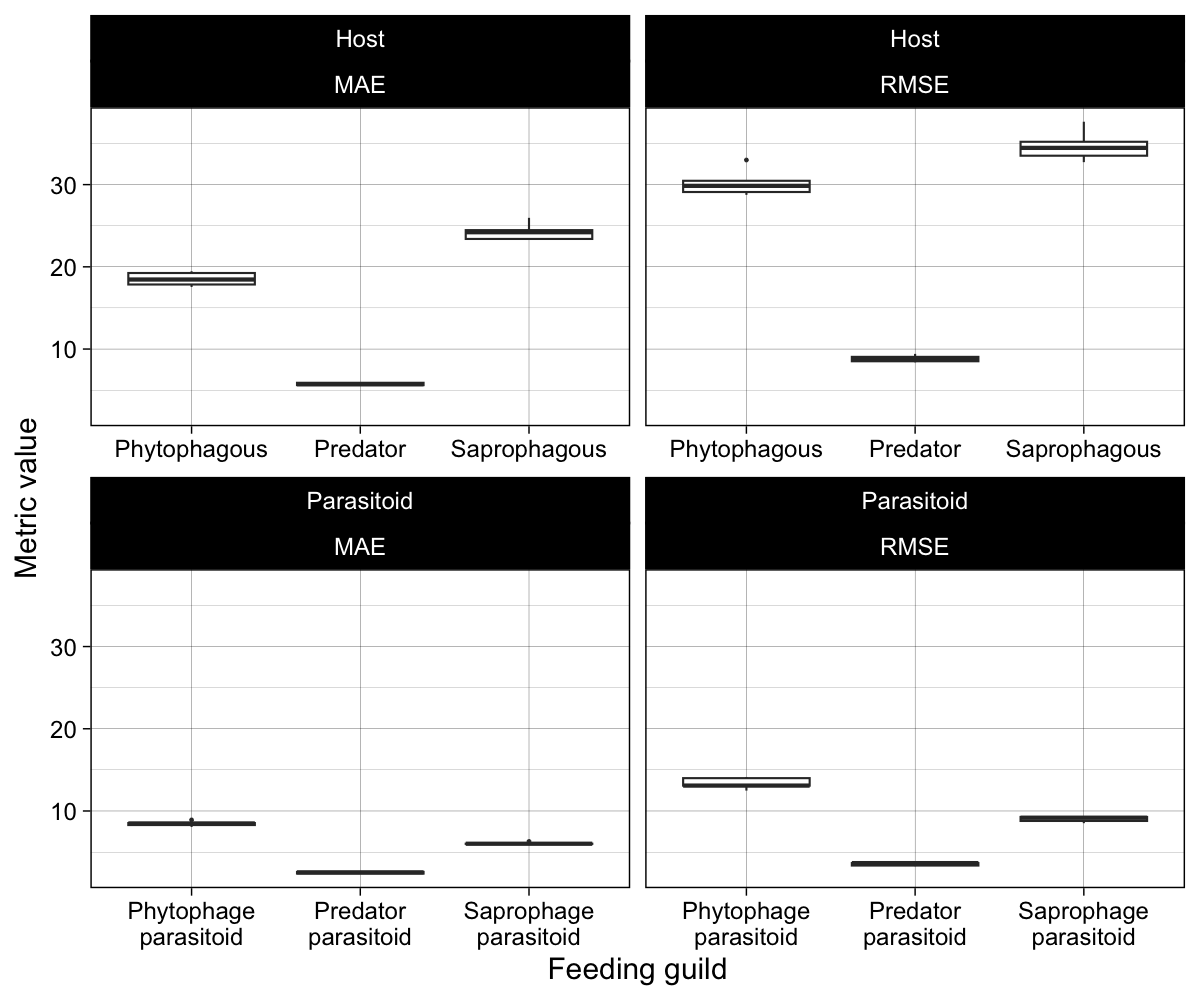


**Figure S3**. Mean absolute error (MAE) and root mean square error (RMSE), for each feeding guild, calculated from 5-fold cross validation. 5-fold cross validation demonstrates relatively good predictive performance across guilds, with all guilds illustrating low variance in predictive error between folds. Guilds with higher Average richness’s (Phytophages, and Saprophages), demonstrate higher predictive errors, but this is proportional considering the total richness of these guilds. Higher root mean error scores (RMSE) are present for most guilds, indicating that there are larger errors that are being penalised more severely.


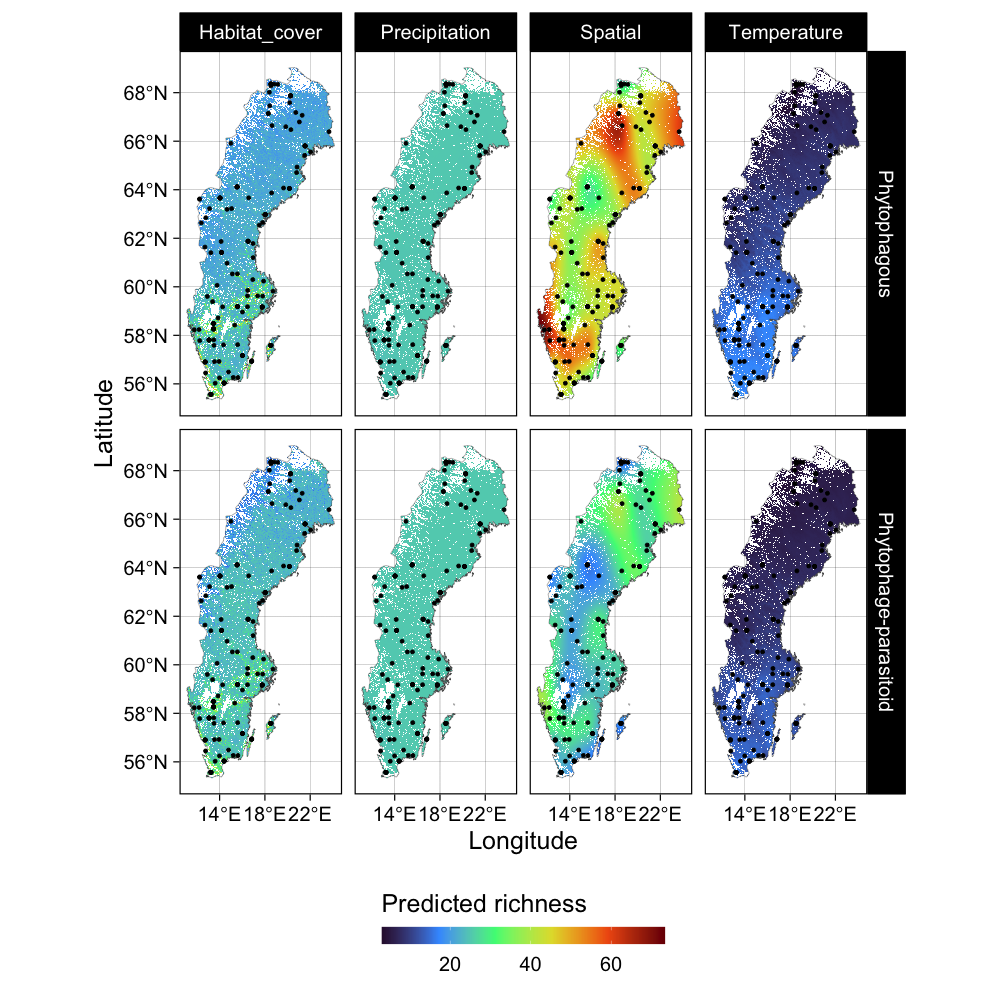


**Figure S4.** The impact of spatially varying model terms on the species richness of Phytophagous insects (top row) and their parasitoids (bottom row). The figure displays predicted species richness for temperature, precipitation, the spatial interaction term, and habitat cover covariates. All non-focal variables are set to 0, so that only the impact of the focal variable on the spatial patterns of guild species richness are visualised. Predictions for the temporally varying terms (precipitation and temperature), represent the average July values for those covariates. Habitat cover represents the sum of all habitat cover covariates included in the model.


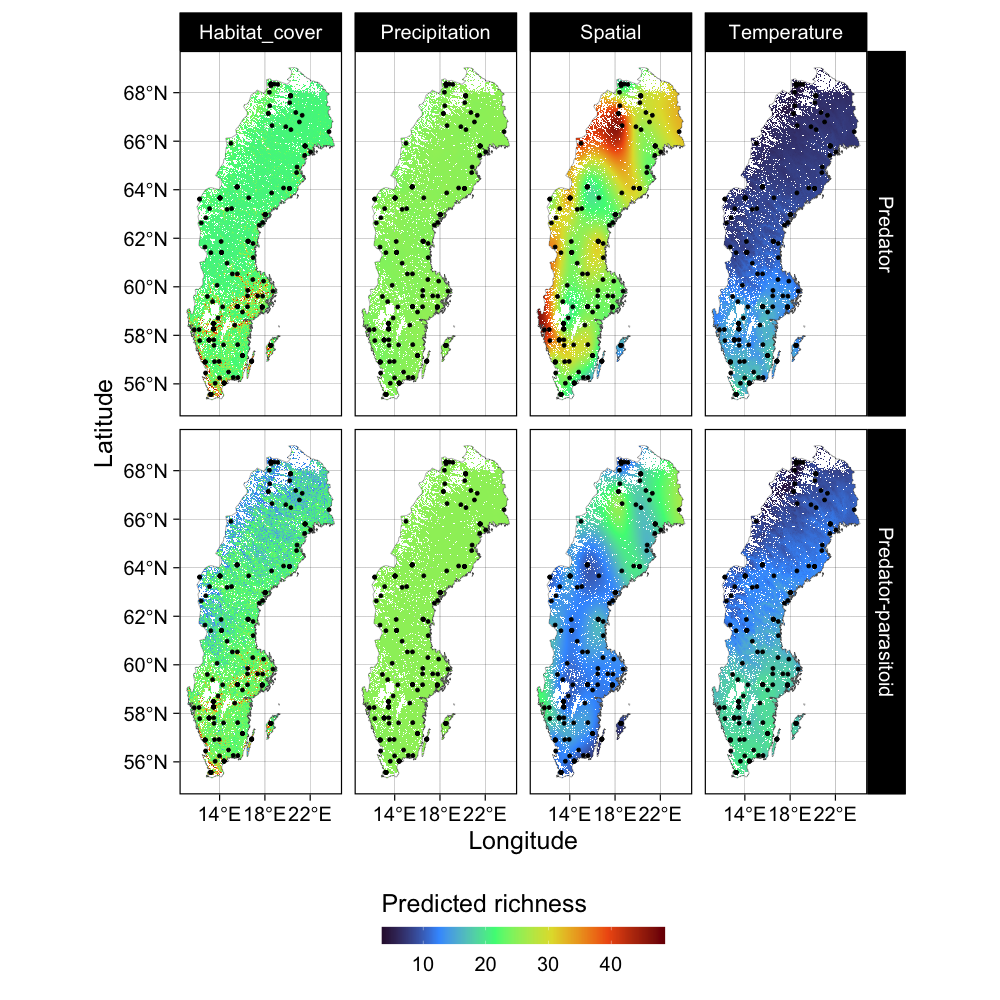


**Figure S5.** The impact of spatially varying model terms on the species richness of Predatory insects (top row) and their parasitoids (bottom row). The figure displays predicted species richness for temperature, precipitation, the spatial interaction term, and habitat cover covariates. All non-focal variables are set to 0, so that only the impact of the focal variable on the spatial patterns of guild species richness are visualised. Predictions for the temporally varying terms (precipitation and temperature), represent the average July values for those covariates. Habitat cover represents the sum of all habitat cover covariates included in the model.


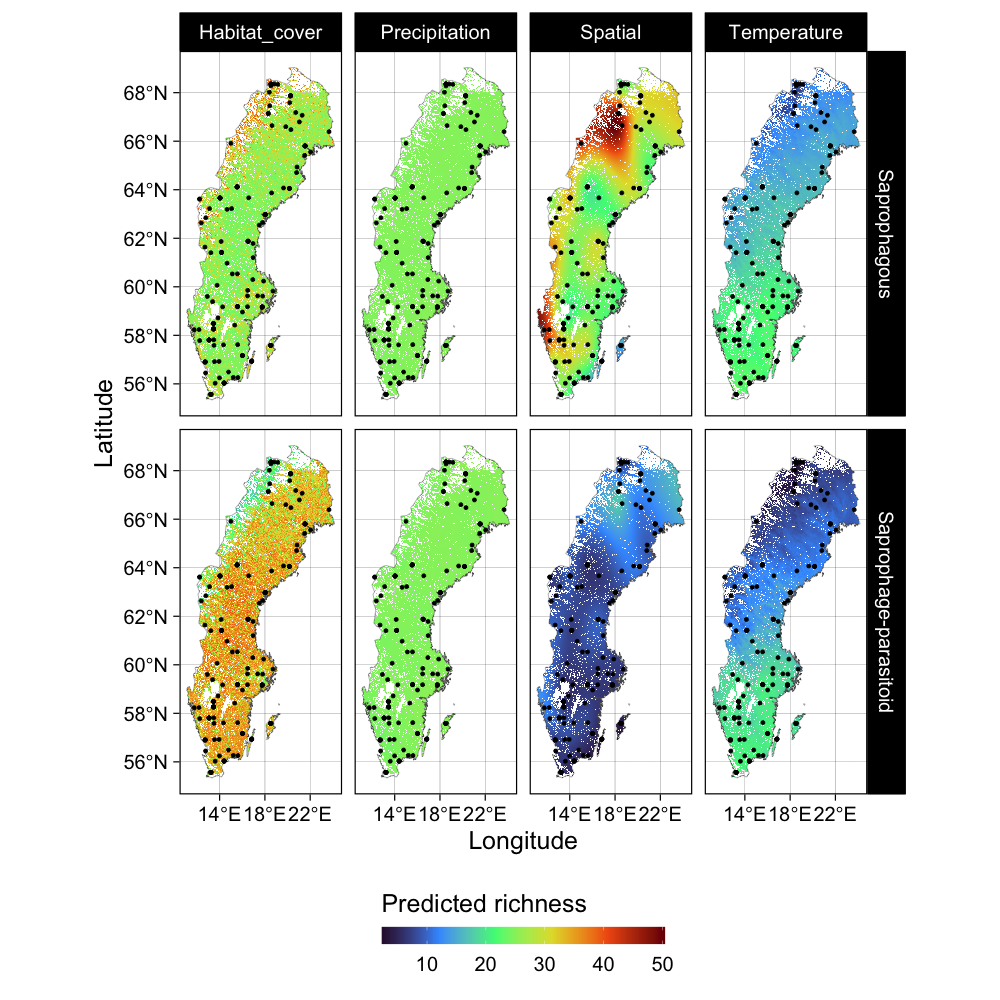


**Figure S6**. The impact of spatially varying model terms on the species richness of Saprophagous insects (top row) and their parasitoids (bottom row). The figure displays predicted species richness for temperature, precipitation, the spatial interaction term, and habitat cover covariates. All non-focal variables are set to 0, so that only the impact of the focal variable on the spatial patterns of guild species richness are visualised. Predictions for the temporally varying terms (precipitation and temperature), represent the average July values for those covariates. Habitat cover represents the sum of all habitat cover covariates included in the model.

**
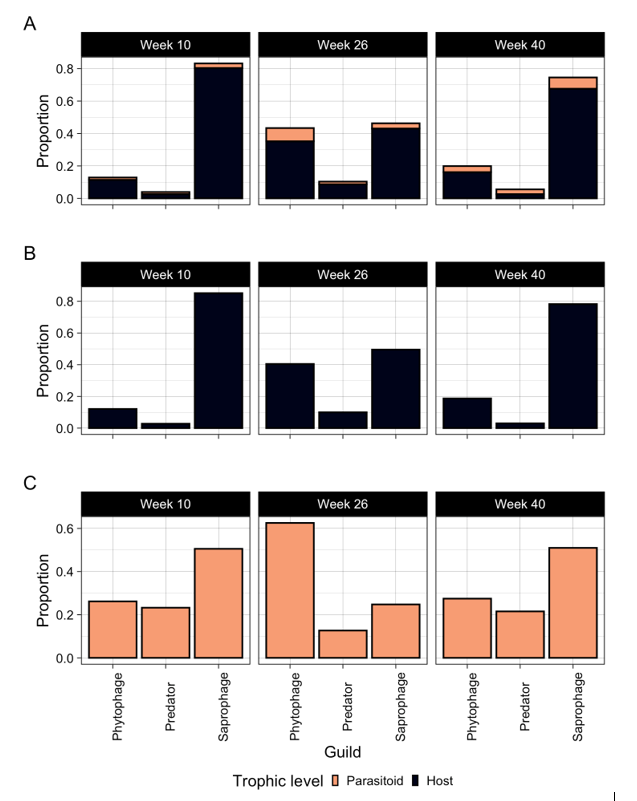
**

**Figure S7**. The predicted composition of insect communities in terms of feeding guild richness throughout the year. The figure shows the proportion of organisms in each guild for all guild-pairs(A), and across hosts only (B), and across parasitoids only (C) at three time points during the year representing the start (week 10), peak (week 26), and end (week 40) of the growing season. As all other predictors are kept constant, this figure represents the average effect across all sites. See table S3 for numerical proportions for each of the guilds.

**
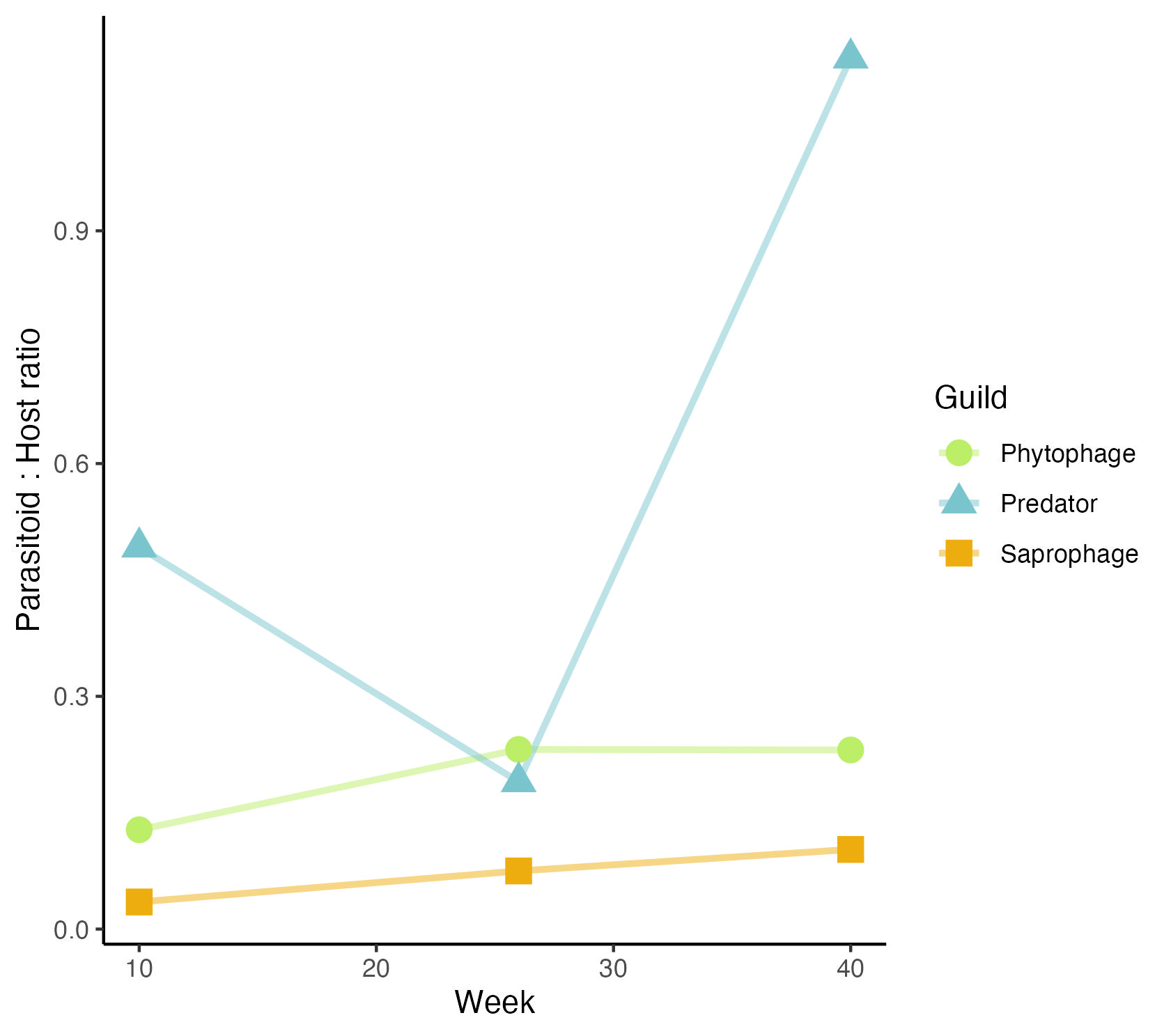
**

**Figure S8.** Parasitoid : host ratios of species richness for each of the guild-pairs, at three time points during the year (week 10, 26 and 40, representing the start, peak and end of the growing season respectively). For raw proportional values of all guilds, see table S3.


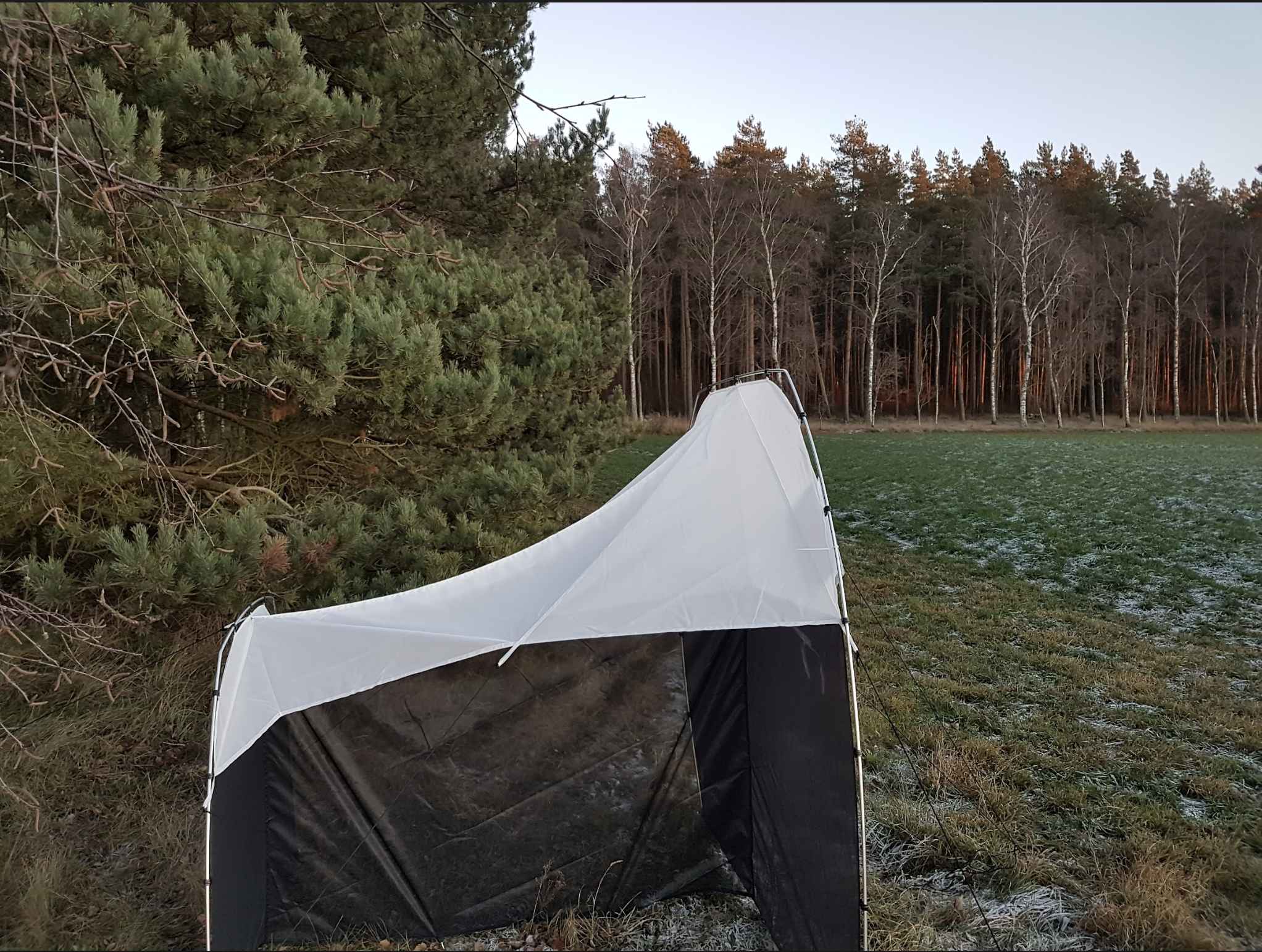


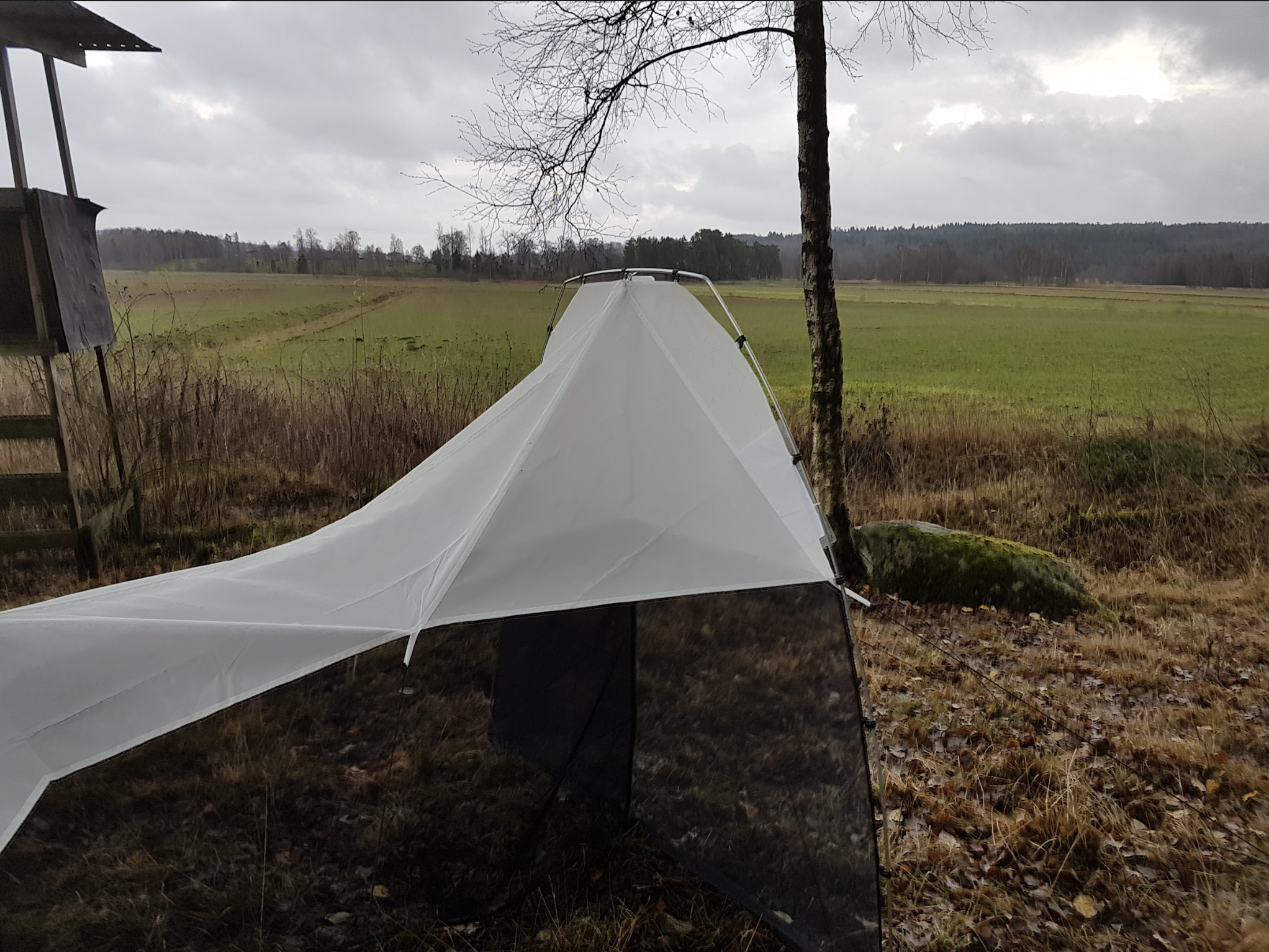

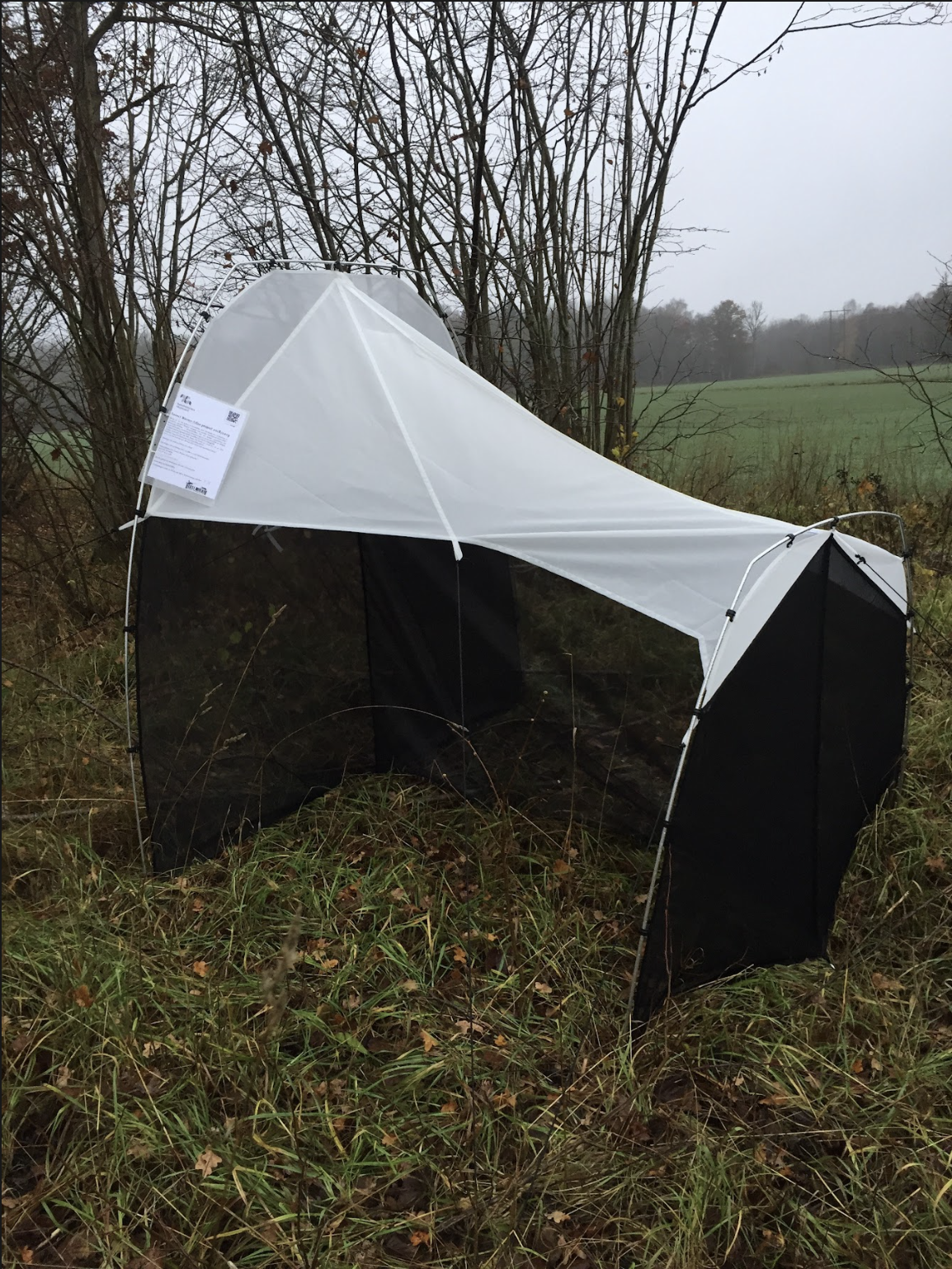


**Figure S9.** Three examples of traps placed next to croplands. In Sweden, agriculture is relatively extensive and croplands are intermixed with forests in the landscape.

**Supplemental references**

Allan, E., Manning, P., Alt, F., Binkenstein, J., Blaser, S., Blüthgen, N., Böhm, S., Grassein, F., Hölzel, N., Klaus, V. H., Kleinebecker, T., Morris, E. K., Oelmann, Y., Prati, D., Renner, S. C., Rillig, M. C., Schaefer, M., Schloter, M., Schmitt, B., … Fischer, M. (2015). Land use intensification alters ecosystem multifunctionality via loss of biodiversity and changes to functional composition. *Ecology Letters*, *18*(8), 834–843. https://doi.org/10.1111/ele.12469

Baselga, A. (2010). Partitioning the turnover and nestedness components of beta diversity. *Global Ecology and Biogeography*, *19*(1), 134–143. https://doi.org/10.1111/j.1466-8238.2009.00490.x

Byrnes, J. E. K., Gamfeldt, L., Isbell, F., Lefcheck, J. S., Griffin, J. N., Hector, A., Cardinale, B. J., Hooper, D. U., Dee, L. E., & Emmett Duffy, J. (2014). Investigating the relationship between biodiversity and ecosystem multifunctionality: Challenges and solutions. *Methods in Ecology and Evolution*, *5*(2), 111–124. https://doi.org/10.1111/2041-210X.12143

Guzman, L. M., Trzcinski, M. K., Barberis, I. M., Céréghino, R., Srivastava, D. S., Gilbert, B., Pillar, V. D., de Omena, P. M., MacDonald, A. A. M., Corbara, B., Leroy, C., Ospina Bautista, F., Romero, G. Q., Kratina, P., Debastiani, V. J., Gonįalves, A. Z., Marino, N. A. C., Farjalla, V. F., Richardson, B. A., … Montero, G. (2021). Climate influences the response of community functional traits to local conditions in bromeliad invertebrate communities. *Ecography*, *44*(3), 440–452. https://doi.org/10.1111/ecog.05437

Ronquist, F., Forshage, M., Häggqvist, S., Karlsson, D., Hovmöller, R., Bergsten, J., Holston, K., Britton, T., Abenius, J., Andersson, B., Buhl, P. N., Coulianos, C.-C., Fjellberg, A., Gertsson, C.-A., Hellqvist, S., Jaschhof, M., Kjærandsen, J., Klopfstein, S., Kobro, S., … Gärdenfors, U. (2020). Completing Linnaeus’s inventory of the Swedish insect fauna: Only 5,000 species left? *PLOS ONE*, *15*(3), e0228561. https://doi.org/10.1371/journal.pone.0228561

Srivastava, D. S., MacDonald, A. A. M., Pillar, V. D., Kratina, P., Debastiani, V. J., Guzman, L. M., Trzcinski, M. K., Dézerald, O., Barberis, I. M., de Omena, P. M., Romero, G. Q., Ospina-Bautista, F., Marino, N. A. C., Leroy, C., Farjalla, V. F., Richardson, B. A., Gonçalves, A. Z., Corbara, B., Petermann, J. S., … Céréghino, R. (2023). Geographical variation in the trait-based assembly patterns of multitrophic invertebrate communities. *Functional Ecology*, *37*(1), 73–86. https://doi.org/10.1111/1365-2435.14096
